# A single-arm prospective phase I trial of ^68^Ga-PFBC01 PET/CT for multiple myeloma B cell maturation antigen imaging

**DOI:** 10.1172/JCI207391

**Published:** 2026-06-30

**Authors:** Tingfei Gu, Zhao Chen, Bo Tang, Tianyao Wang, Qi Yang, Huihui Liu, Zeyin Liang, Qian Wang, Yang Zhang, Yuhua Sun, Mingyi Di, Tingting Yuan, Yongkang Qiu, Yimeng Du, Lele Song, Shengnan Wu, Wei Wang, Xiaojie Xu, Yujun Dong, Lei Kang

**Affiliations:** 1 Department of Nuclear Medicine and; 2 Department of Hematology, Peking University First Hospital, Beijing, China.; 3 Department of Genetic Engineering, Beijing Institute of Biotechnology, Beijing, China.; 4 Department of Pathology, Peking University First Hospital, Beijing, China.

**Keywords:** Clinical Research, Hematology, Oncology, Diagnostic imaging

## Abstract

**BACKGROUND:**

B cell maturation antigen (BCMA) is a key therapeutic target in multiple myeloma (MM), yet its whole-body in vivo distribution and role in disease assessment remain incompletely defined. We aimed to evaluate the safety, diagnostic performance, and clinical utility of a novel BCMA-targeted PET tracer, ^68^Ga-PFBC01, in patients with plasma cell disorders.

**METHODS:**

We conducted a single-center, prospective, single-arm phase I trial (ClinicalTrials.gov NCT06717113). Fifty patients underwent ^68^Ga-PFBC01 PET/CT, including 40 with paired ^18^F-FDG PET/CT for head-to-head comparison. Primary outcomes included diagnostic performance (sensitivity, specificity, PPV, NPV, and interreader agreement). Secondary outcomes included correlations with clinical biomarkers, treatment response assessment, impact on clinical decision-making, and safety.

**RESULTS:**

^68^Ga-PFBC01 PET/CT demonstrated superior diagnostic performance compared with ^18^F-FDG PET/CT (sensitivity 96.9% versus 84.6%; specificity 71.4% versus 60.0%). Quantitative PET-derived tumor burden correlated with M protein (*R* = 0.325, *P* = 0.026), free light chains (*R* = 0.340–0.437, *P* ≤ 0.015), soluble BCMA (*R* = 0.433, *P* = 0.050), and bone marrow plasma cells (*R* = 0.682, *P* < 0.001). Imaging findings altered clinical management in multiple cases, enabling both therapy escalation and deescalation. Blood-pool uptake strongly correlated with soluble BCMA (*R* = 0.899, *P* < 0.001) and overall disease burden (*R* = 0.736, *P* < 0.001). No serious tracer-related adverse events were observed; 2 patients (4%) experienced mild events.

**CONCLUSION:**

^68^Ga-PFBC01 PET/CT provides biologically specific, whole-body assessment of MM, outperforming ^18^F-FDG and enabling integrated evaluation of tumor burden and systemic disease activity, with direct implications for clinical decision-making.

**TRIAL REGISTRATION:**

ClinicalTrials.gov NCT06717113.

**FUNDING:**

National Natural Science Foundation of China (82472018, 82402320), Beijing Nova Program (20240484725), National High Level Hospital Clinical Research Funding (Interdisciplinary Research Project of Peking University First Hospital, 2024IR07, Scientific and Technological Achievements Transformation Incubation Guidance Fund Project of Peking University First Hospital, 2025CX38, 2024CX18). Research Achievement Transformation Project of Peking University First Hospital, 2025ZH02), Clinical Medicine Plus X - Young Scholars Project of Peking University, the Fundamental Research Funds for the Central Universities (PKU2026PKULCXQ038).

## Abstract 2

BCMA-targeted PET/CT provided biologically specific imaging of multiple myeloma, improving diagnosis, response evaluation, and assessment of systemic disease burden.

## Graphical abstract

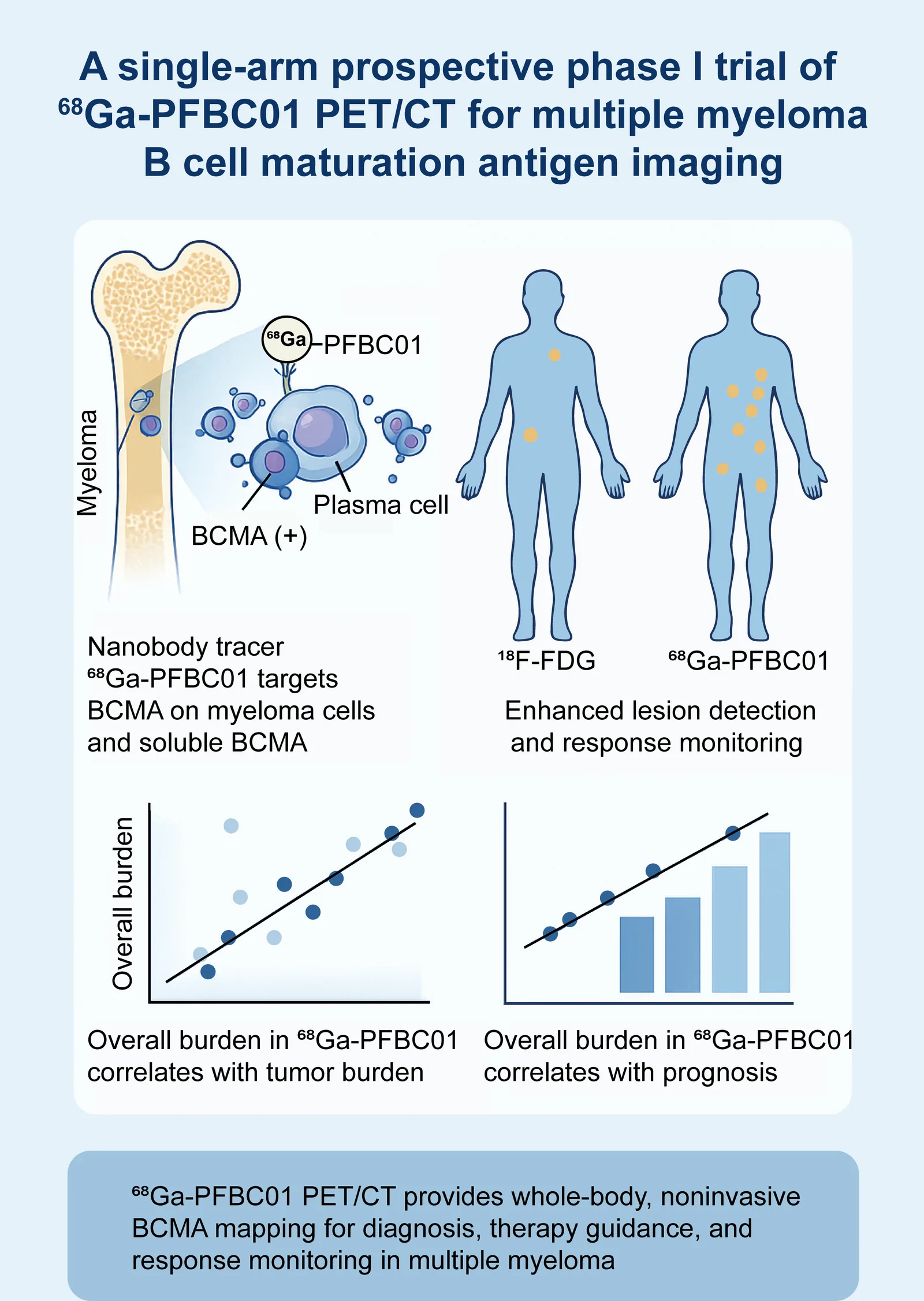

## Introduction

Multiple myeloma (MM) is a biologically and clinically heterogeneous plasma-cell malignancy characterized by clonal plasma cell expansion (1). Despite major therapeutic advances, disease evaluation remains largely dependent on serological markers (2) and bone marrow sampling (3). These approaches provide limited spatial information and may fail to capture the full extent of a systemic and heterogeneous disease, leaving clinicians without a comprehensive in vivo assessment of tumor burden and distribution.

Molecular imaging with ^18^F-fluorodeoxyglucose (^18^F-FDG) PET/CT has improved the evaluation of hematologic malignancies (4, 5); however, FDG uptake in MM varies widely due to differences in hexokinase-2 expression, resulting in false negatives in 11%–33% of patients (6–9). In addition, nonspecific uptake in inflammatory or reparative processes can confound interpretation (10). As such, FDG PET/CT does not fully reflect myeloma-specific biology and has limited utility in guiding targeted therapy or accurately assessing treatment response.

B cell maturation antigen (BCMA) has emerged as a highly specific marker of malignant plasma cells (11, 12) and a central therapeutic target in MM (13, 14). Its restricted expression profile makes it an attractive candidate for molecular imaging, enabling direct visualization of disease biology. In this context, we previously reported the first-in-human feasibility of BCMA-targeted PET/CT imaging, demonstrating its potential for specific detection of myeloma lesions (15). However, the clinical utility of BCMA-targeted imaging across the disease continuum, including diagnosis, tumor burden assessment, treatment guidance, response evaluation, and prognostic stratification, has not been systematically established.

Here, we conducted a prospective clinical trial to comprehensively evaluate the performance of a nanobody-based BCMA PET tracer, ^68^Ga-PFBC01, in patients with plasma cell disorders. Our aim was to define its role as an integrated imaging biomarker for whole-body disease characterization and clinical decision making in multiple myeloma.

## Results

### In vitro characterization.

**Figure 1:**
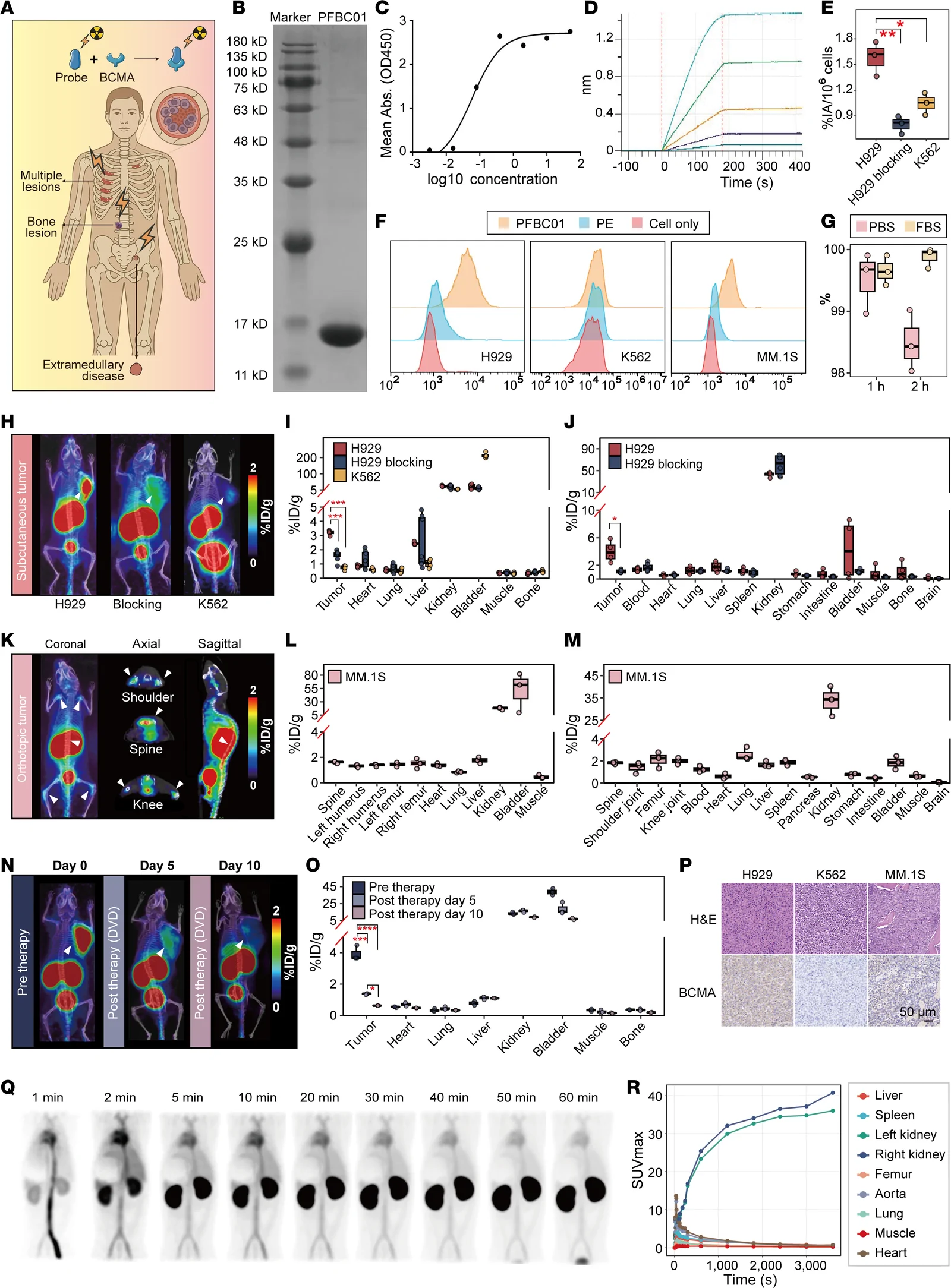
Preparation and preclinical characterization of ^68^Ga-PFBC01 for PET/CT imaging. (**A**) Schematic illustration of the ^68^Ga-PFBC01 PET/CT probe for the precise diagnosis of MM. (**B**) SDS-PAGE confirmed greater than 90% purity of the sdAb following Ni^2+^ affinity purification. (**C**) ELISA assay confirmed the high binding affinity of the PFBC01 for the BCMA protein. (**D**) Bio-layer interferometry showed strong binding affinity between PFBC01 and BCMA protein. (**E**) Cell uptake and blocking assays demonstrated specific binding in H929 cells compared with blocking and K562 cells (*n* = 3). (**F**) Flow cytometry analysis showing rightward fluorescence shift in BCMA-positive H929 and MM.1S cells. (**G**) TLC analysis showed high radiochemical purity in PBS and FBS (*n* = 3). (**H**) Maximum intensity projection (MIP) PET images of subcutaneous tumor models. (**I**) Quantitative Region of Interest (ROI) analysis of PET imaging in subcutaneous tumor models (*n* = 4 for H929; *n* = 7 for H929 blocking; *n* = 4 for K562). (**J**) Biodistribution study at 1 hour after injection corroborating the PET findings of subcutaneous tumor models. (**K**) Representative PET images (coronal, axial, and sagittal views) of an orthotopic MM.1S model. The probe efficiently accumulated in various tumor foci (white arrows), clearly delineating even small lesions. (**L**) Quantitative PET analysis of the orthotopic model (*n* = 3). (**M**) Biodistribution analysis at 1 hour after injection of the orthotopic model (*n* = 3). (**N**) MIP PET images for monitoring therapy response (DVD regimen). A reduction in tumor uptake was evident on day 5 after treatment, with a further decrease observed on day 10. (**O**) Quantitative PET analysis of therapy response (*n* = 3). (**P**) Histopathology and IHC confirmed strong BCMA expressions in H929 and MM.1S tumors, but minimal staining in K562 tumors. (**Q**) Dynamic PET imaging following intravenous injection of the probe in a healthy cynomolgus monkey. (**R**) Quantitative PET analysis in healthy cynomolgus monkeys showed rapid clearance and predominant renal excretion of the tracer (*n* = 3).

Preclinical studies established the binding specificity, stability, and imaging performance of ^68^Ga-PFBC01. A BCMA-specific single-domain antibody (sdAb) library was generated through alpaca immunization followed by standard molecular cloning procedures, including RNA extraction, PCR amplification, ligation, and bacterial transformation (Figure 1A). SDS-PAGE confirmed successful purification of the sdAb, yielding a single protein band with purity exceeding 90% following Ni^2+^ affinity chromatography (Figure 1B). ELISA and biolayer interferometry confirmed high affinity and specificity toward recombinant BCMA (Figure 1, C and D). In vitro assays demonstrated selective uptake of ^68^Ga-PFBC01 in BCMA-positive H929 cells (1.58 ± 0.20 %IA/10^6^ cells), significantly exceeding that in H929 blocking cells (0.80 ± 0.11 %IA/10^6^ cells, *P* = 0.009) and BCMA-negative K562 cells (1.05 ± 0.13 %IA/10^6^ cells, *P* = 0.024; Figure 1E). Flow cytometry further validated these findings, showing strong fluorescence shifts in BCMA-positive lines but not in K562 cells (Figure 1F). ^68^Ga-PFBC01 exhibited excellent radiochemical stability (> 98%) in PBS and FBS over 4 hours (Figure 1G and Supplemental Figure 1; supplemental material available online with this article; https://doi.org/10.1172/JCI207391DS1).

### In vivo imaging and biodistribution.

In subcutaneous xenograft models, ^68^Ga-PFBC01 PET/CT showed intense uptake in BCMA-positive H929 tumors but minimal signal in blocking and BCMA-negative controls (Figure 1H). Quantitative region-of-interest (ROI) analysis showed tumor uptake of 3.21 ± 0.18 % percentage of injected dose per gram of tissue) (ID/g) in H929 mice, significantly higher than in H929-blocking (1.60 ± 0.38 %ID/g) and K562 (0.70 ± 0.24 %ID/g) groups (Figure 1I). Ex vivo biodistribution at 1 hour after injection corroborated the PET findings (4.00 ± 1.55 %ID/g in the H929 model versus 1.12 ± 0.24 %ID/g in the H929-blocking group, *P* = 0.033; Figure 1J). In orthotopic MM.1S models, ^68^Ga-PFBC01 PET/CT clearly demonstrated multifocal skeletal lesions (Figure 1K), ranging from 1.33 ± 0.11 to 1.61 ± 0.09 %ID/g across skeletal sites (Figure 1L), consistent with ex vivo measurements (Figure 1M).

To assess treatment monitoring capability, ^68^Ga-PFBC01 PET was used to evaluate response to the DVD (daratumumab, bortezomib, and dexamethasone) chemotherapy (16). Tumor uptake markedly declined by day 5 and further by day 10 from 3.93 ± 0.47 to 0.63 ± 0.00 %ID/g; Figure 1, N and O). IHC staining confirmed high BCMA expression in H929 and MM.1S tumors (Figure 1P).

In healthy cynomolgus monkeys, dynamic PET revealed rapid blood clearance (peak SUVmax = 13.7 ± 1.2 at 35 s) and renal excretion, with predominant uptake in kidneys (peak SUVmax = 36–41 at 3,600 s; Figure 1, Q and R). Based on dynamic PET imaging in healthy cynomolgus monkeys, preliminary dosimetry estimates of ^68^Ga-PFBC01 were calculated using the OLINDA/EXM software package (Version 2.2; Vanderbilt University, Nashville, Tennessee, USA), and the results are summarized in Supplemental Table 1.

### Clinical investigation of ^68^Ga-PFBC01 PET/CT.

**Figure 2:**
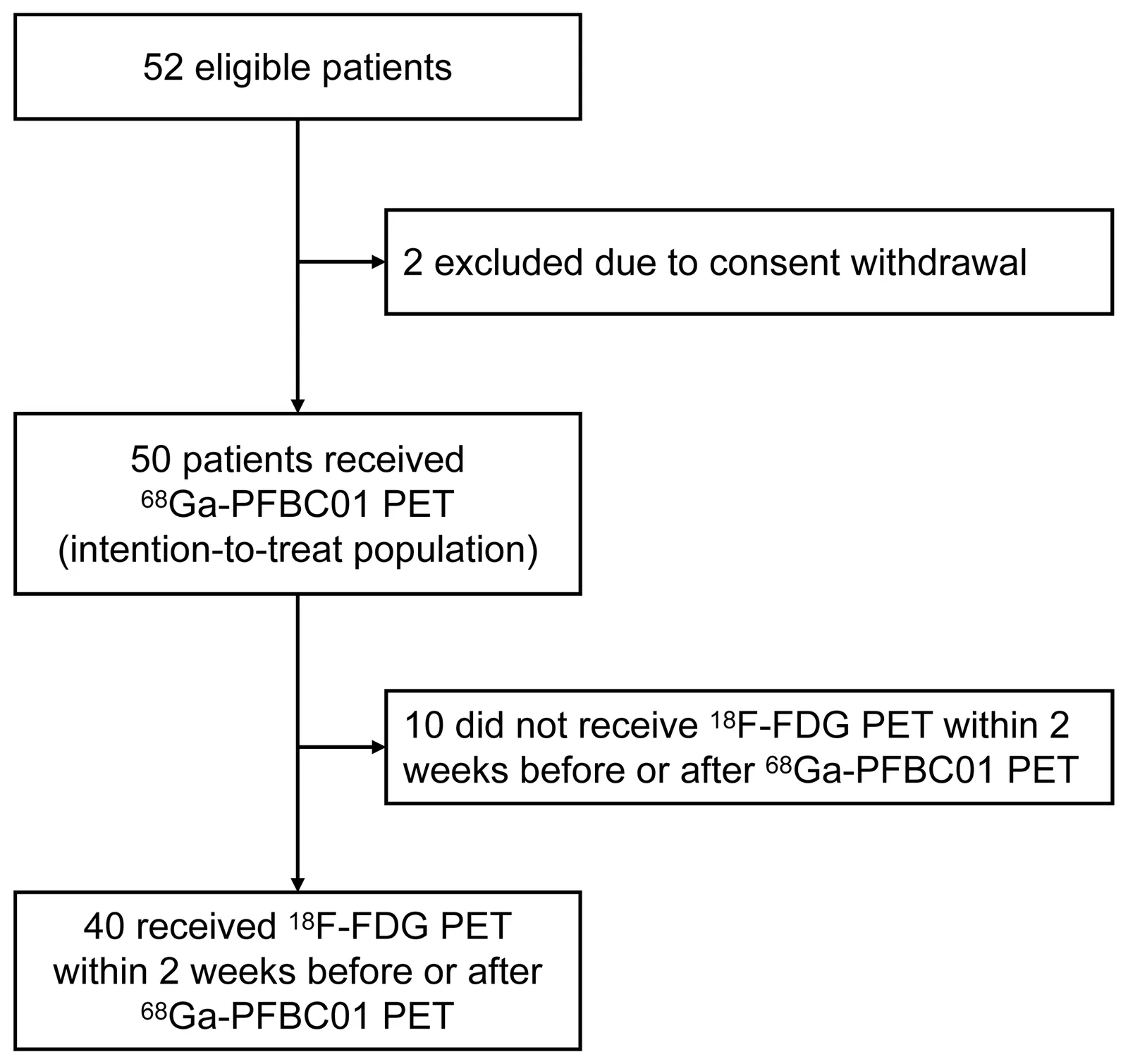
CONSORT — Consolidated Standards of Reporting Trials — diagram.

Between April 21, 2025, and September 4, 2025, a total of 52 eligible patients were screened for participation in this single-center, single-arm Phase I trial (Figure 2). Among them, 2 individuals withdrew consent prior to imaging. Consequently, 50 patients underwent ^68^Ga-PFBC01 PET/CT. Of these, 40 patients also underwent ^18^F-FDG PET/CT within a 2-week window before or after the ^68^Ga-PFBC01 scan, allowing for direct comparison between the 2 imaging modalities (Supplemental Table 2). All 50 patients had histopathological confirmation of multiple myeloma, ensuring diagnostic accuracy for subsequent analyses. Baseline characteristics are summarized in Supplemental Table 3.

### Pathological Correlation of ^68^Ga-PFBC01 PET/CT.

**Figure 3:**
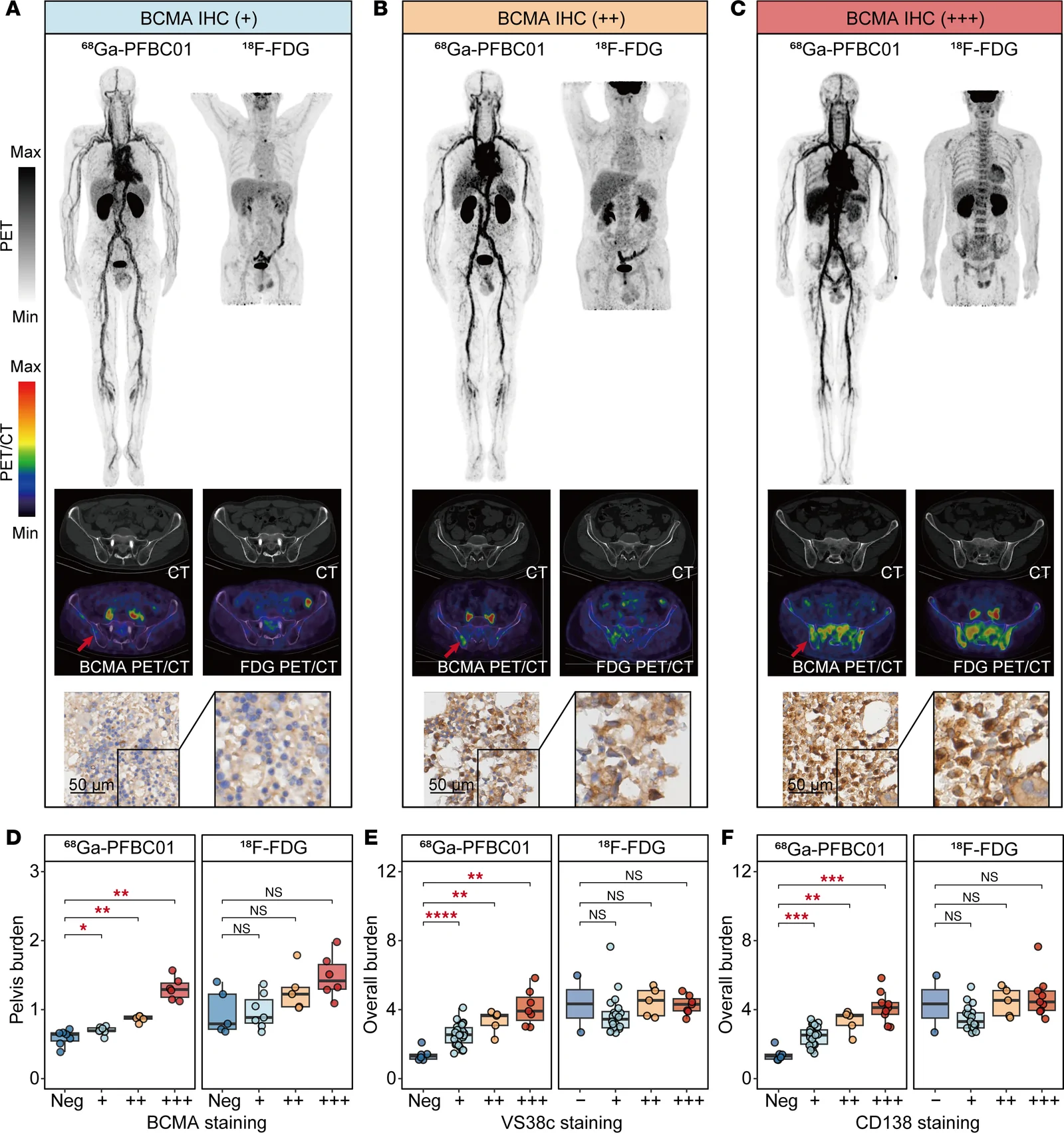
Pathological Correlation of ^68^Ga-PFBC01 PET/CT and ^18^F-FDG PET/CT with BCMA Expression in newly diagnosed patients with MM. (**A**) Head-to-head comparison of ^68^Ga-PFBC01 PET/CT and ^18^F-FDG in a patient with weak BCMA expression (+). (**B**) Head-to-head comparison of ^68^Ga-PFBC01 PET/CT and ^18^F-FDG in a patient with moderate BCMA expression (++). (**C**) Head-to-head comparison of ^68^Ga-PFBC01 PET/CT and ^18^F-FDG in a patient with strong BCMA expression (+++). (**D**) Head-to-head comparison of ^68^Ga-PFBC01 PET/CT and ^18^F-FDG in BCMA immunohistochemical staining. (**E**) Head-to-head comparison of ^68^Ga-PFBC01 PET/CT and ^18^F-FDG in Vs38c immunohistochemical staining. (**F**) Head-to-head comparison of ^68^Ga-PFBC01 PET/CT and ^18^F-FDG in CD138 immunohistochemical staining.

To minimize observer bias, a deep learning–based algorithm was used to automatically segment skeletal structures on CT into thoracic, spinal, and pelvic regions and calculated regional SUVmean values to derive the overall burden (Supplemental Figure 2). Uptake of ^68^Ga-PFBC01 PET/CT showed a strong correlation with BCMA immunohistochemical expression, suggesting the binding specificity of ^68^Ga-PFBC01 to BCMA. Signal intensity increased progressively from BCMA IHC+ (Figure 3A) to BCMA IHC++ (Figure 3B) and BCMA IHC+++ (Figure 3C) in newly diagnosed patients with MM, a finding that was further confirmed quantitatively (Figure 3D). In addition to BCMA IHC, tracer uptake also correlated significantly with Vs38c (Figure 3E) and CD138 (Figure 3F) staining, supporting the concordance between molecular imaging and histopathologic plasma cell markers.

### Diagnostic performance of ^68^Ga-PFBC01 PET/CT.

**Figure 4:**
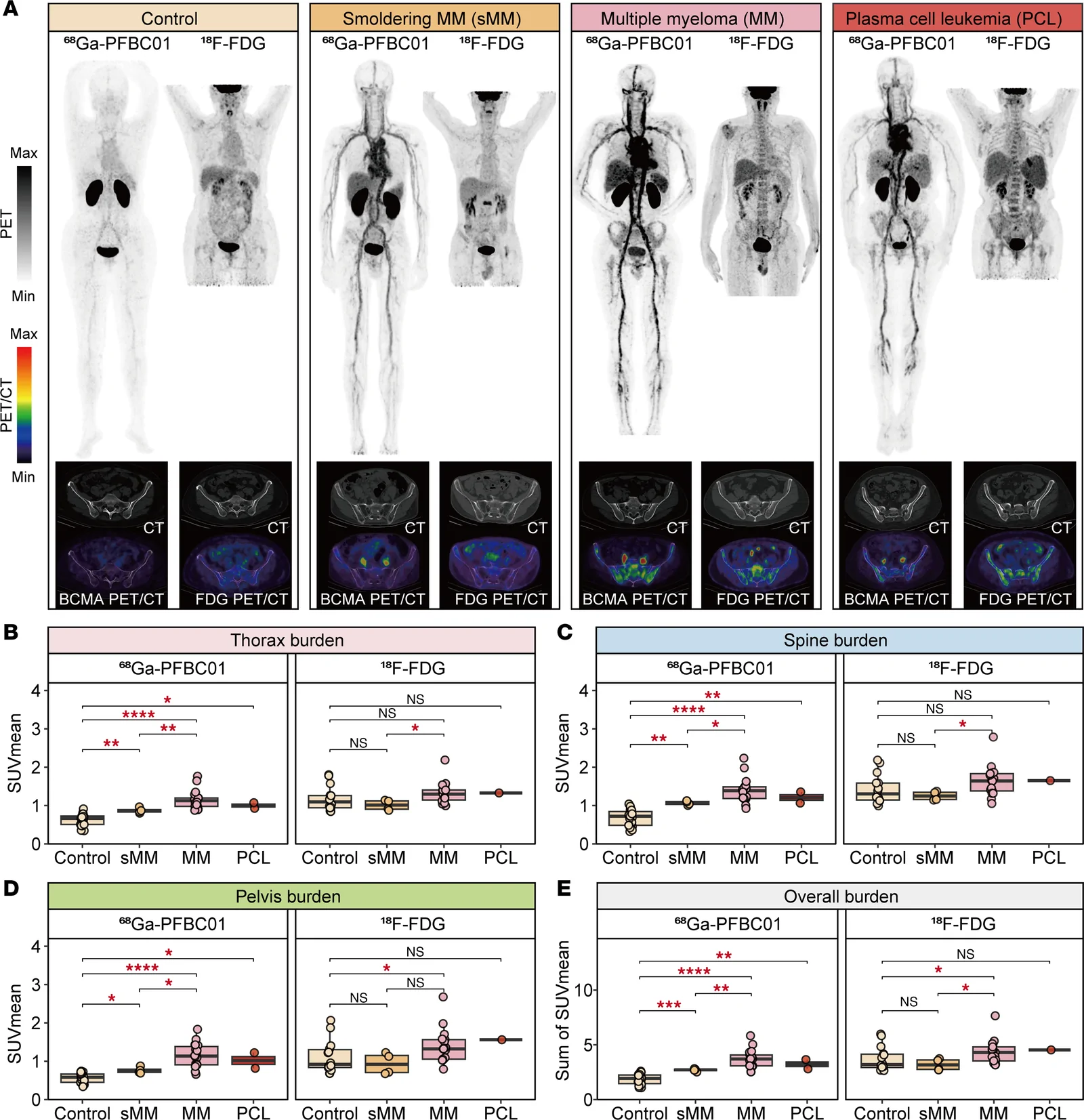
Head-to-head comparison of ^68^Ga-PFBC01 and ^18^F-FDG PET/CT for the diagnosis of plasma cell disorders. (**A**) Representative ^68^Ga-PFBC01 and ^18^F-FDG PET/CT images in patients with different stages of plasma cell disorders. From left to right: healthy control, smoldering multiple myeloma (SMM), multiple myeloma (MM), and plasma cell leukemia (PCL). (**B**) Quantitative comparison of thorax burden between ^68^Ga-PFBC01 and ^18^F-FDG PET/CT. (**C**) Quantitative comparison of spine burden between ^68^Ga-PFBC01 and ^18^F-FDG PET/CT. (**D**) Quantitative comparison of pelvis burden between ^68^Ga-PFBC01 and ^18^F-FDG PET/CT. (**E**) Quantitative comparison of overall burden between ^68^Ga-PFBC01 and ^18^F-FDG PET/CT.

In diagnostic assessment, ^68^Ga-PFBC01 PET/CT clearly differentiated among control, smoldering multiple myeloma (sMM), MM, and plasma cell leukemia (PCL, Figure 4A). High lesion-to-background contrast was observed in the thoracic bones (Figure 4B), spine (Figure 4C), and pelvis (Figure 4D) — regions most frequently involved in MM — allowing site-specific disease detection in cases where ^18^F-FDG PET/CT failed to distinguish pathological from normal uptake. When integrated on a whole-body basis, ^68^Ga-PFBC01 PET/CT achieved more accurate stratification across the spectrum of plasma cell disorders, whereas ^18^F-FDG PET/CT was only able to distinguish controls from MM without discriminating intermediate states such as sMM or PCL (Figure 4E).

### Clinical decision-making guided by ^68^Ga-PFBC01 PET/CT.

**Figure 5:**
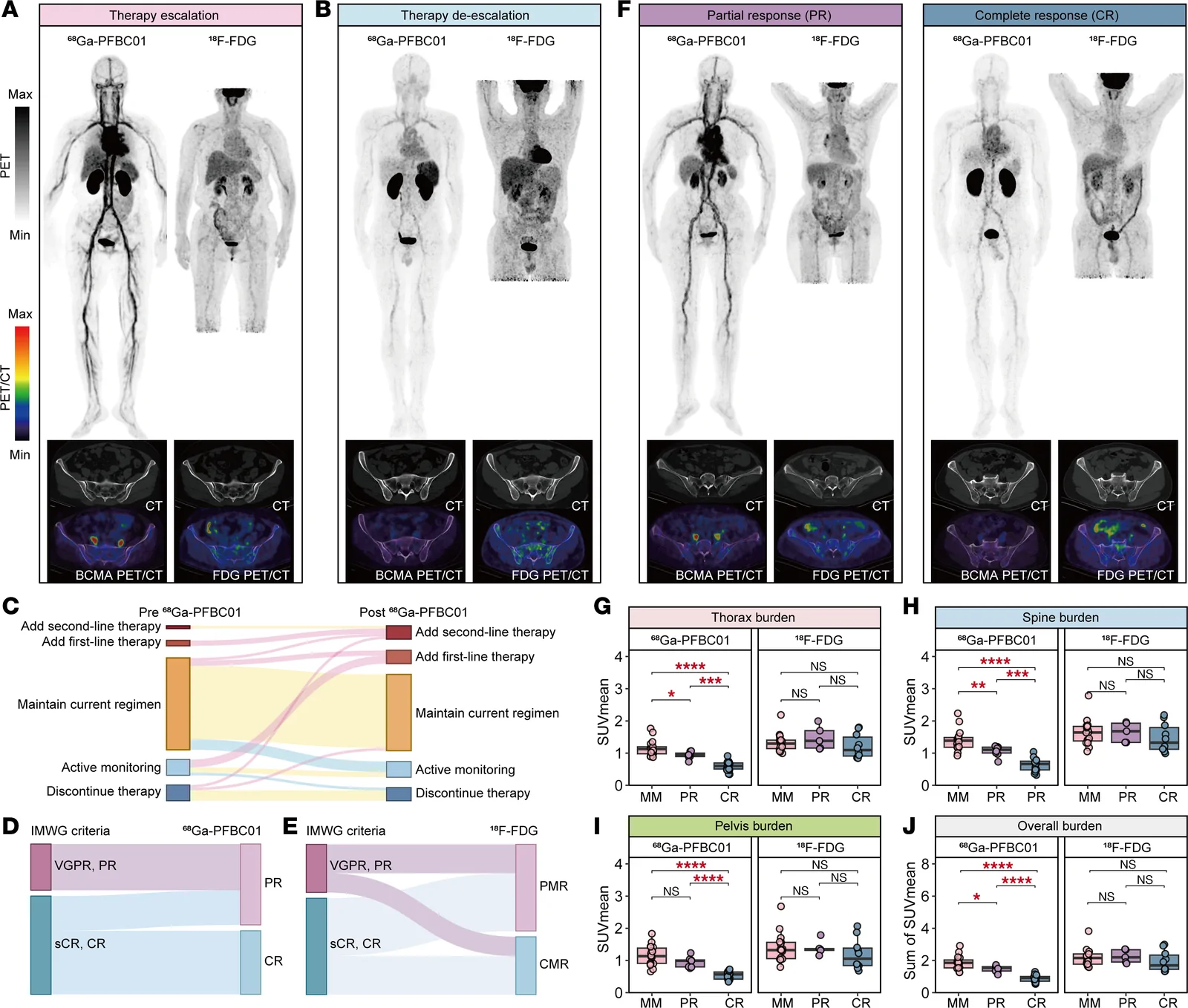
Head-to-head comparison of ^68^Ga-PFBC01 and ^18^F-FDG PET/CT for therapy guidance and response assessment in multiple myeloma. (**A**) Representative PET/CT images of patients who underwent therapy escalation based on ^68^Ga-PFBC01 findings. (**B**) Representative PET/CT images of patients who underwent therapy deescalation based on ^68^Ga-PFBC01 findings. (**C**) Summary of therapy decisions guided by ^68^Ga-PFBC01. (**D**) Comparison between serological response according to the International Myeloma Working Group (IMWG) criteria and PET/CT response assessed by ^68^Ga-PFBC01. (**E**) Comparison between serological IMWG response and PET/CT response assessed by ^18^F-FDG. (**F**) Representative ^68^Ga-PFBC01 and ^18^F-FDG PET/CT images acquired after treatment in patients with multiple myeloma, from left to right: patient achieved partial response (PR) and patient achieved complete response (CR). (**G**) Quantitative comparison of thorax burden between ^68^Ga-PFBC01 and ^18^F-FDG PET/CT during treatment evaluation. (**H**) Quantitative comparison of spine burden between ^68^Ga-PFBC01 and ^18^F-FDG PET/CT during treatment evaluation. (**I**) Quantitative comparison of pelvis burden between ^68^Ga-PFBC01 and ^18^F-FDG PET/CT during treatment evaluation. (**J**) Quantitative comparison of overall burden between ^68^Ga-PFBC01 and ^18^F-FDG PET/CT during treatment evaluation.

^68^Ga-PFBC01 PET/CT demonstrated clear clinical utility in guiding therapeutic decisions. In the first representative case, a patient with sMM showed intense ^68^Ga-PFBC01 uptake, leading to treatment escalation from active monitoring (initially determined based on ^18^F-FDG PET/CT and clinical history) to the addition of first-line therapy (Figure 5A). In contrast, another patient with treated MM exhibited residual glucose metabolism on ^18^F-FDG PET/CT and was initially planned to continue maintenance therapy. However, ^68^Ga-PFBC01 PET/CT revealed no significant residual lesions, prompting treatment deescalation to active monitoring, which was later supported by MRD negativity (Figure 5B). A comprehensive treatment-flow Sankey diagram (Figure 5C) further illustrates the impact of ^68^Ga-PFBC01 on clinical decision making. Among patients with solitary plasmacytoma (*n* = 2), ^18^F-FDG PET/CT suggested continuation of the current regimen after surgical resection, whereas persistent BCMA-targeted uptake on ^68^Ga-PFBC01 prompted escalation to add first-line radiotherapy. Two patients with sMM transitioned from active monitoring to first-line therapy based on strong ^68^Ga-PFBC01 uptake. Two patients with extramedullary disease (EMD) escalated from first-line to second-line therapy due to specific ^68^Ga-PFBC01 avid lesions. One newly diagnosed patient with MM similarly escalated from active monitoring to first-line therapy. Conversely, 4 posttreatment patients with MM who exhibited residual ^18^F-FDG uptake but no detectable ^68^Ga-PFBC01 lesions underwent therapy deescalation from maintenance to active monitoring. Additionally, 1 treated patient with MM who achieved strict complete response (sCR) showed no ^68^Ga-PFBC01 uptake, supporting treatment discontinuation.

### Treatment response evaluation in ^68^Ga-PFBC01 PET/CT.

Further comparison of PET-based response evaluation according to IMWG criteria revealed strong concordance between ^68^Ga-PFBC01 PET/CT and clinical outcomes. Among 8 patients with VGPR or PR, all were categorized as PR, while 17 patients with sCR or CR were classified as CR (*n* = 11) or PR (*n* = 6) on ^68^Ga-PFBC01 PET/CT (Figure 5D). In contrast, ^18^F-FDG PET/CT demonstrated lower discriminative accuracy: among 5 patients with VGPR or PR, 3 were rated as partial metabolic response (PMR) and 2 as complete metabolic response (CMR); among 10 patients with sCR or CR, only 4 were categorized as CMR and 6 as PMR (Figure 5E).

For response visualization, ^68^Ga-PFBC01 PET/CT clearly differentiated disease states at PR, and CR (Figure 5F). High lesion-to-background contrast was consistently preserved across thoracic bones (Figure 5G), spine (Figure 5H), and pelvis (Figure 5I). In contrast, ^18^F-FDG PET/CT, reflecting glucose metabolism, often displayed nonspecific posttreatment uptake, reducing its ability to distinguish response phases regionally or in overall burden assessment. In whole-body analyses, ^68^Ga-PFBC01 PET/CT showed superior sensitivity and specificity for discriminating remission states compared with ^18^F-FDG PET/CT, which frequently overestimated metabolic activity in posttherapy lesions (Figure 5J).

### Tumor burden and prognostic evaluation in ^68^Ga-PFBC01 PET/CT.

**Figure 6:**
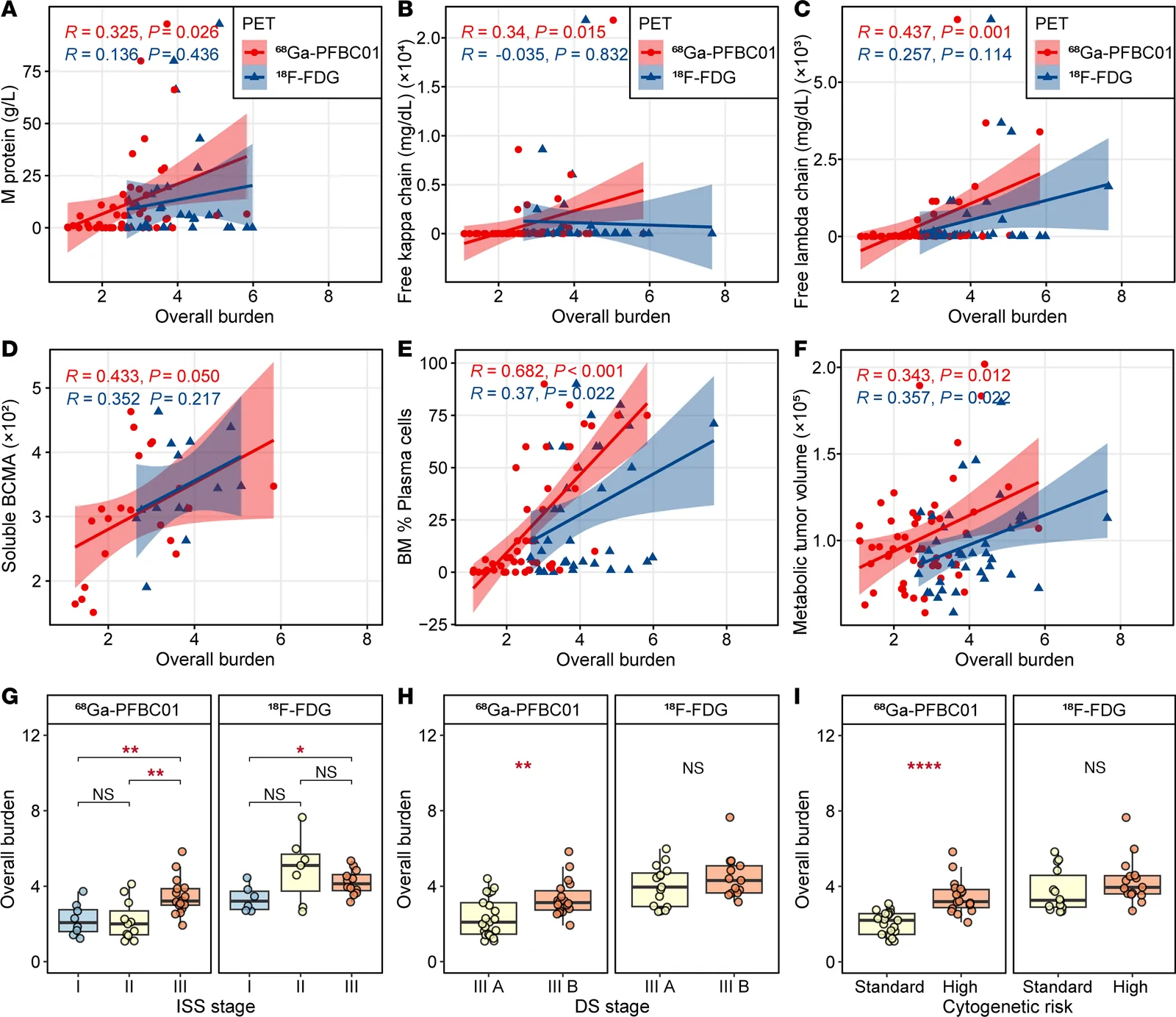
Head-to-head comparison of ^68^Ga-PFBC01 and ^18^F-FDG PET/CT for tumor burden evaluation and prognostic assessment in multiple myeloma. (**A**) Correlation between PET/CT–derived overall burden and M protein (g/L). (**B**) Correlation between PET/CT–derived overall burden and free κ chain (mg/dL). (**C**) Correlation between PET/CT–derived overall burden and free λ chain (mg/dL). (**D**) Correlation of ^68^Ga-PFBC01 and ^18^F-FDG PET/CT–derived overall burden with serum soluble BCMA levels. (**E**) Correlation between PET/CT–derived overall burden and bone marrow plasma cell percentage. (**F**) Correlation between PET/CT–derived overall burden and metabolic tumor volume (MTV). (**G**) Prognostic evaluation based on International Staging System (ISS) stage. (**H**) Prognostic evaluation based on Durie–Salmon (DS) stage. (**I**) Prognostic evaluation according to cytogenetic risk.

Quantitative analysis further revealed that ^68^Ga-PFBC01 PET/CT provided an accurate reflection of tumor burden. The total PET intensity correlated positively with M protein level (*R* = 0.325, *P* = 0.026, Figure 6A), free κ light chain (*R* = 0.340, *P* = 0.015, Figure 6B), free λ light chain (*R* = 0.437, *P* = 0.001, Figure 6C), soluble BCMA (*R* = 0.433, *P* = 0.050, Figure 6D), bone marrow plasma cell percentage (*R* = 0.682, *P* < 0.001, Figure 6E), and metabolic tumor volume (*R* = 0.343, *P* = 0.012, Figure 6F). Tracer uptake also stratified prognostic subgroups, distinguishing International Scoring System (ISS) stage III from stages I (*P* = 0.004) and II (*P* = 0.006, Figure 6G), Durie–Salmon (DS) stage III A from III B (*P* = 0.006, Figure 6H), and standard from high-risk cytogenetic profiles (*P* < 0.001, Figure 6I). These findings indicate that ^68^Ga-PFBC01 PET/CT captures disease biology reflected in both tumor burden and prognosis.

### Diagnostic accuracy and interreader consistency of ^68^Ga-PFBC01.

**Figure 7:**
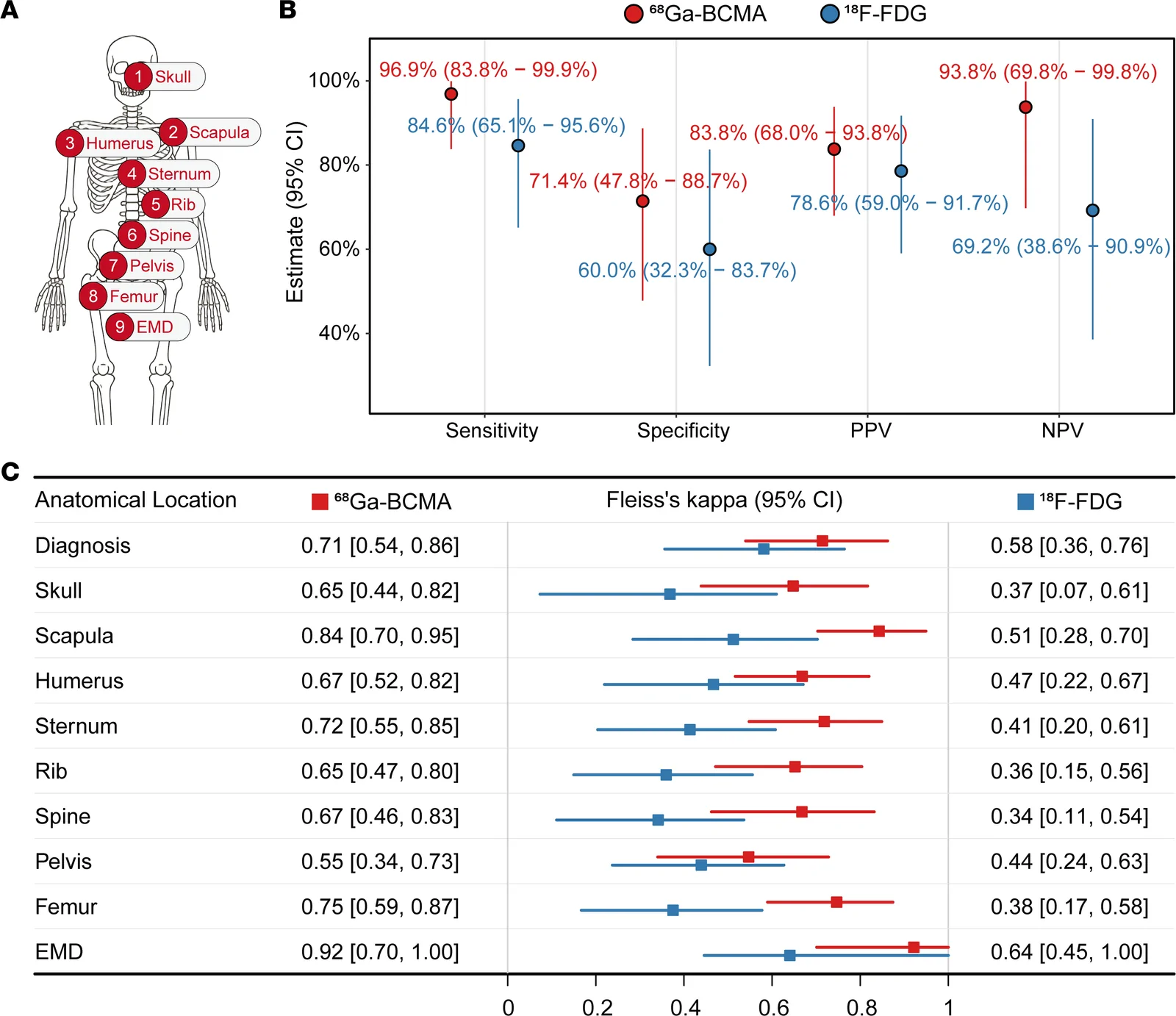
Evaluation of diagnostic performance between ^68^Ga-PFBC01 and ^18^F-FDG PET/CT. (**A**) Schematic diagram of anatomical regions evaluated for image interpretation, including the skull, scapula, humerus, sternum, rib, spine, pelvis, femur, and extramedullary disease (EMD). (**B**) Head-to-head comparison of diagnostic performance between ^68^Ga-PFBC01 and ^18^F-FDG PET/CT, from left to right: sensitivity, specificity, positive predictive value (PPV), and negative predictive value (NPV). (**C**) Comparison of interreader consistency between ^68^Ga-PFBC01 and ^18^F-FDG PET/CT across diagnostic categories and anatomical sites.

In head-to-head regional comparisons across 9 anatomic compartments — skull, scapula, humerus, sternum, ribs, spine, pelvis, femur, and EMD — ^68^Ga-PFBC01 PET/CT consistently outperformed ^18^F-FDG PET/CT (Figure 7A). On a per-patient basis, sensitivity [96.9% (83.8%–99.9%) versus 84.6% (65.1%–95.6%)], specificity [71.4% (47.8%–88.7%) versus 60.0% (32.3%–83.7%)], positive predictive value (PPV) [83.8% (68.0%–93.8%) versus 78.6% (59.0%–91.7%)], and negative predictive value (NPV) [93.8% (69.8%–99.8%) versus 69.2% (38.6–%90.9%)] were all higher for ^68^Ga-PFBC01 PET/CT (Figure 7B). Interreader agreement analysis using Fleiss’ κ showed improved consistency for ^68^Ga-PFBC01 PET/CT across both whole-body diagnosis and site-specific evaluations compared with ^18^F-FDG PET/CT (Figure 7C).

### Blood-pool signal as a biomarker of systemic disease activity.

An unexpected yet biologically meaningful finding was the high blood-pool uptake of ^68^Ga-PFBC01 PET/CT, distinct from the rapid clearance typically observed with nanobody-based tracers (17). Automated segmentation and quantification of blood-pool SUVmean (Supplemental Figure 3A) were calculated. Blood-pool SUVmean increased progressively with disease severity (Supplemental Figure 3B), demonstrating strong association with treatment response categories (Supplemental Figure 3C). Moreover, blood-pool activity correlated with prognostic indices, including ISS stage (Supplemental Figure 3D), DS stage (Supplemental Figure 3E), and cytogenetic risk classification (Supplemental Figure 3F), as well as with overall disease burden (*R* = 0.736, *P* < 0.001; Supplemental Figure 3G), bone marrow plasma cell percentage (*R* = 0.545, *P* < 0.001; Supplemental Figure 3H), and CD138 staining (Supplemental Figure 3I). Plasma-soluble BCMA levels were measured by indirect ELISA (Supplemental Figure 3J) and correlated positively with blood pool SUVmean (*R* = 0.899, *P* < 0.001; Supplemental Figure 3K). These results suggest that blood-pool activity may serve as a surrogate imaging biomarker for circulating BCMA load, providing an integrated assessment of systemic disease activity.

### Safety evaluation of ^68^Ga-PFBC01 PET/CT.

In 2 (4%) of 50 participants, 2 adverse events were classified as possibly related to ^68^Ga-PFBC01 PET/CT within 30 days (Supplemental Table 4), including nausea (*n* = 1) and dizziness (*n* = 1).

## Discussion

This phase I clinical trial demonstrates that ^68^Ga-PFBC01 PET/CT enables precise, noninvasive visualization of disease burden across the full clinical spectrum of plasma cell disorders. Compared with ^18^F-FDG PET/CT, this imaging modality exhibited superior diagnostic accuracy, therapy guidance capability, treatment-response sensitivity, and prognostic relevance, addressing several long-standing unmet needs in the management of MM.

Soluble BCMA concentrations have been shown to increase progressively from MGUS to sMM and active MM, reflecting the biological continuum of plasma cell proliferation (18, 19). In this study, ^68^Ga-PFBC01 PET/CT transformed these biochemical findings into whole-body imaging evidence, allowing precise differentiation among control, sMM, MM, and PCL. While ^18^F-FDG PET/CT remains the recommended standard for MM evaluation, its dependence on glucose metabolism limits sensitivity in lesions with low hexokinase-2 expression (5). In contrast, ^68^Ga-PFBC01 PET/CT provided higher lesion contrast within the thoracic bones, spine, and pelvis — regions commonly affected in MM — allowing site-specific disease detection where ^18^F-FDG failed to discriminate disease states without whole-body integration. To minimize observer bias, a deep learning–based algorithm was used to automatically segment the skeleton, heart, and kidneys and to extract SUVmean values, ensuring consistent and objective quantification (20).

This improved diagnostic performance naturally raises the question of whether ^68^Ga-PFBC01 PET/CT can also quantify disease burden more accurately. Quantitative assessment is particularly relevant for patients with relapsed or refractory MM, who often develop oligosecretory or extramedullary disease not adequately captured by traditional laboratory markers or focal bone marrow biopsies (21). Consistent with prior findings that soluble BCMA reflects plasma cell burden and prognosis (19, 21, 22), we observed strong correlations between PET-derived overall burden and metabolic tumor volume with soluble BCMA levels, bone marrow plasma cell percentage, and other established prognostic indices. Together, these findings suggest that BCMA-targeted PET/CT bridges the gap between serologic and morphologic assessment, offering a truly whole-body, quantitative measure of disease activity. Such quantification not only refines tumor burden assessment but may also facilitate prediction of immune-related toxicities (23) and suboptimal therapeutic responses (24).

Beyond diagnostic and quantitative performance, an equally important implication of ^68^Ga-PFBC01 PET/CT lies in its ability to guide individualized clinical decision making. In this trial, imaging findings directly influenced therapeutic choices across multiple clinical scenarios — from therapy escalation in smoldering or extramedullary myeloma to deescalation or treatment discontinuation in patients achieving deep remission. These imaging-driven decisions, later validated by MRD assessments, illustrate how BCMA-targeted PET/CT could bridge diagnostic precision with actionable clinical management. Such capability supports its integration into personalized treatment algorithms, potentially optimizing therapy intensity and minimizing both overtreatment and undertreatment in MM.

Having established its diagnostic, tumor burden quantitative and therapy-guidance utility, we next evaluated ^68^Ga-PFBC01 PET/CT for treatment-response monitoring. Conventional laboratory assays, including serum or urine electrophoresis and free light chain measurements, fail in up to 12% of patients with oligosecretory or nonsecretory disease, a situation increasingly encountered after multiple lines of therapy (25). These limitations frequently lead to diagnostic delays or exclusion from clinical trials (26). By contrast, as BCMA is rapidly shed from plasma cells into circulation (27), BCMA-based assessments may capture dynamic changes earlier than M-protein or free light chain (27). Moreover, unlike β2-micro globulin or light chain clearance, which are affected by renal function (28), ^68^Ga-PFBC01 PET/CT lesion uptake remained stable across varying renal parameters, consistent with prior reports that soluble BCMA levels are independent of renal impairment (22, 29). In our trial, this biological stability translated into clear clinical advantages. ^68^Ga-PFBC01 PET/CT successfully differentiated patients between active newly diagnosed MM, PR, and CR, outperforming ^18^F-FDG PET/CT in sensitivity and reproducibility. Although more than half of patients with MM achieve CR following initial therapy (30), those with minimal residual disease (MRD) continue to face relapse risk (31). Current International Myeloma Working Group guidelines recommend next-generation flow cytometry (NGF), next-generation sequencing (NGS), and ^18^F-FDG PET/CT for MRD detection (5), yet each approach has certain drawbacks. NGF and NGS are invasive and subject to sampling bias (32), whereas ^18^F-FDG PET/CT may yield false negatives in low-metabolic (6–9) and false positives in inflammation or posttreatment effects (33, 34). By contrast, ^68^Ga-PFBC01 PET/CT overcomes these challenges by providing a noninvasive, quantitative, and reproducible whole-body evaluation. Previously, we reported the first-in-human BCMA targeted PET/CT tracer with pilot results (15), combined with head-to-head comparison with NGF and NGS, confirming that BCMA-targeted PET imaging can serve as a reliable modality for both molecular and clinical endpoints in longitudinal therapy monitoring.

An intriguing and distinctive observation in this study was the unusually high blood pool uptake of the tracer, which is contrary to the rapid clearance typically seen with nanobody-based agents (16, 35). This finding likely reflects binding between circulating soluble BCMA and the radiolabeled nanobody, resulting in systemic signal retention. Consistent with this interpretation, preliminary pharmacokinetic modeling analysis in healthy nonhuman primates demonstrated low radiation dose estimates to the blood pool, suggesting that the elevated blood-pool signal observed in patients was unlikely to be explained solely by prolonged intravascular retention (Supplemental Figure 4). Importantly, blood pool SUVmean correlated positively with tumor burden, disease stage, and soluble BCMA levels, suggesting that this parameter itself may serve as an imaging method of systemic BCMA burden. These findings also suggest that elevated blood-pool activity should be carefully considered during image interpretation, particularly in patients with high circulating soluble BCMA levels or advanced disease burden. Thus, ^68^Ga-PFBC01 PET/CT might provide information related to both lesion uptake and circulating BCMA in a single scan. This approach offers practical advantages over conventional ELISA or flow cytometry, which require multistep procedures and longer turnaround times.

Elevated renal uptake observed in this study is consistent with the known pharmacokinetics of nanobody tracers, which undergo glomerular filtration and tubular reabsorption. Previous BCMA-targeted imaging studies, such as those by Wei et al. (36) and Thomas et al. (37), demonstrated modest tumor uptake and high renal accumulation, raising concerns about nephrotoxicity. ^89^Zr-DFO-BCMAh230430 exhibited high tumor uptake but nonspecific bone accumulation (38), likely related to species-specific metabolism in murine models (39). By contrast, ^68^Ga-PFBC01 showed favorable pharmacokinetics, safety, and biodistribution across subcutaneous, orthotopic, and nonhuman primate models, and crucially achieved successful clinical translation in humans. Although renal signal intensity warrants careful interpretation, it is clinically manageable given the low frequency of direct renal infiltration in MM (40). To further evaluate the potential influence of renal function on tracer biodistribution, we performed exploratory analyses of kidney uptake in relation to renal functional parameters. Kidney SUVmean showed a negative correlation with serum creatinine and a positive correlation with estimated glomerular filtration rate (eGFR, MDRD equation), suggesting that renal uptake might reflect preserved physiologic tracer clearance rather than nonspecific retention associated with renal impairment. Moreover, patients with renal damage demonstrated significantly lower renal tracer uptake (Supplemental Figure 5). These findings support the interpretation that the elevated renal signal might reflect the expected pharmacokinetic behavior of nanobody-based tracers undergoing glomerular filtration and tubular handling.

Despite these encouraging findings, several limitations should be acknowledged. This was a single-center, phase I study with a modest sample size, and subgroup analyses across different disease states should therefore be interpreted cautiously. Treatment-response evaluation also relied primarily on cross-sectional rather than longitudinal comparisons. In addition, because this trial was conducted at a single center in China, all enrolled participants self identified as Asian, which may limit the generalizability of the findings to broader racial and ethnic populations. Although most paired ^18^F-FDG PET/CT and ^68^Ga-PFBC01 PET/CT scans were completed within 5 days without interval antimyeloma treatment, residual temporal variability between imaging studies cannot be fully excluded. A forthcoming multicenter, prospective study will address these limitations by incorporating paired pre- and posttreatment scans, implementing more tightly synchronized imaging intervals, enrolling patients across different disease states and demographic backgrounds, and integrating MRD assessment (NGF and NGS) and survival outcomes to further define the clinical role of ^68^Ga-PFBC01 PET/CT in MM management.

In summary, ^68^Ga-PFBC01 PET/CT enables whole-body, noninvasive quantification of BCMA expression, outperforms ^18^F-FDG PET/CT in diagnostic precision, disease burden assessment, therapy guidance, and response monitoring. By integrating signals from both solid and circulating disease components, this platform establishes a unified biomarker that bridges serologic and tumor burden in multiple myeloma. These findings extend the role of PET imaging beyond metabolism toward target-specific disease mapping, offering a foundation for precision diagnosis, therapy selection, and response monitoring in BCMA-directed treatment paradigms.

## Methods

For studies performed in primates and mice, see Supplemental Data*, Methods for studies performed in primates and mice*.

### Sex as a biological variable

Sex was not considered as a biological variable in this study. Both male and female participants and animal models were included; however, the study was not designed or powered to evaluate sex-specific differences, and no stratified analyses by sex were performed.

### Preparation and characterization of BCMA-specific nanobody PFBC01

This study developed a nanobody with high affinity for BCMA as the targeting moiety for the radiotracer. The BCMA-specific nanobody PFBC01 was generated by immunizing healthy adult alpacas with recombinant BCMA protein (ACROBiosystems). Single-domain antibody (sdAb, VHH or nanobody) fragments were amplified from lymphocyte RNA, cloned into a phagemid vector, and screened by phage display. The selected clone PFBC01 was expressed in E. coli BL21(DE3) cells, purified via Ni^2+^ affinity chromatography, and analyzed by SDS–PAGE to verify structural integrity and molecular weight.

The binding affinity of PFBC01 to recombinant BCMA protein was assessed by enzyme-linked immunosorbent assay (ELISA) and biolayer interferometry (BLI). For BLI, nanobody-loaded Protein A biosensors were exposed to serial dilutions of BCMA (50–3.125 nM), and kinetic parameters were analyzed using Octet Analysis Studio software.

### Radiolabeling and quality control of 68Ga-PFBC01

PFBC01 was conjugated with the chelator p-SCN-Bn-NOTA (trihydrochloride) at a molar ratio of 1:10–1:20 in carbonate-bicarbonate buffer (pH 9.2) and incubated for 2 hours. The conjugate was purified using PD-10 desalting columns and concentrated by ultrafiltration. For radiolabeling, ^68^Ga eluted from a ^68^Ge/^68^Ga generator was mixed with 200 μg of NOTA-conjugated PFBC01 in sodium acetate buffer (pH 4.5) and incubated at 37°C for 15 minutes. The labeling product was purified using a PD-10 column to remove free gallium ions.

Radiochemical purity was assessed by radio-thin layer chromatography (radio-TLC), and only preparations with purity ≥ 95% were used for further studies. In vitro stability was evaluated in saline and fetal bovine serum (FBS) at 37°C for up to 4 hours.

### Study design

This was a single-center, single-arm, interventional Phase I trial conducted at Peking University First Hospital. All participants self identified as Asian. Race and ethnicity data were self reported by participants at enrollment.

#### Inclusion criteria.

Inclusion criteria included (a) patients with suspected or previously diagnosed multiple myeloma (MM) who were scheduled for bone marrow aspiration or tissue biopsy within 2 weeks, including those undergoing initial diagnostic evaluation or follow-up/reevaluation for disease monitoring or relapse; (b) patients with confirmed symptomatic MM; (c) ability to understand and voluntarily sign written informed consent; (d) ability to comply with study procedures; and (e) Eastern Cooperative Oncology Group (ECOG) performance status ≤ 2.

#### Exclusion criteria.

Exclusion criteria included pregnancy or lactation; inability to comprehend study procedures or cooperate with protocol requirements; or any other condition judged by the investigator to potentially interfere with study participation.

Written informed consent was obtained from all participants before enrollment in the study. The study protocol was approved by the Ethics Committee of Peking University First Hospital (Approval No. 2024600-002) and registered on ClinicalTrials.gov (NCT06717113; https://clinicaltrials.gov/study/NCT06717113?cond=BCMA%20PET&rank=1).

At enrollment, peripheral blood tests performed within one week of ^68^Ga-PFBC01 PET/CT were collected. Quantitative values of M-protein, free κ light chain, and free λ light chain were recorded as indicators of tumor burden. In addition, plasma samples were obtained for indirect ELISA measurement of soluble BCMA levels. Clinical staging parameters, including Durie–Salmon (DS) stage (41), International Scoring System (ISS) stage (42), and cytogenetic risk category (43), were collected for prognostic assessment. Patients classified as DS stage IIIB were defined as having renal impairment.

### Intervention: PET/CT procedures

#### ^18^F-FDG PET/CT Protocol.

Patients fasted for at least 6 hours prior to the examination. ^18^F-FDG (0.05–0.1 mCi/kg) was administered intravenously. Around 1 hour post-injection, imaging from the head to the proximal thigh was performed using a United Imaging uMI 780 PET/CT scanner. Patients were scanned in the supine position under quiet breathing. Images were reconstructed using the OSEM algorithm to generate coronal, sagittal, axial, and fused PET/CT images.

#### ^68^Ga-PFBC01 PET/CT protocol.

The procedure followed a similar workflow as ^18^F-FDG PET/CT without fasting preparation. After intravenous injection of quality-controlled ^68^Ga-PFBC01 (0.03–0.06 mCi/kg), whole-body imaging (head to mid-thigh) was acquired at approximately post-injection 1 hour using the same scanner. Delayed imaging was performed when suspicious lesions were confirmed. Image reconstruction was performed identically using the OSEM method.

### Outcomes

The primary endpoint was to evaluate the diagnostic performance of ^68^Ga-PFBC01 PET/CT for MM detection, analyzed on both per-patient and per-lesion bases. The diagnostic spectrum included smoldering multiple myeloma (sMM), MM, and plasma cell leukemia (PCL). Diagnostic metrics included sensitivity, specificity, positive predictive value (PPV), negative predictive value (NPV), and inter-reader consistency.

Secondary outcomes included the quantitative assessment of overall burden using deep-learning–based skeletal segmentation; the impact of ^68^Ga-PFBC01 PET/CT on clinical decision-making; comparison of treatment-response assessment with IMWG serological criteria and ^18^F-FDG PET/CT; correlations between PET-derived overall burden and serological or pathological markers of disease burden (including M-protein, serum free light chains, bone marrow plasma cell percentage, soluble BCMA, and metabolic tumor volume); prognostic stratification according to ISS stage, Durie–Salmon stage, and cytogenetic risk; and safety. An exploratory endpoint evaluated the association between blood-pool ^68^Ga-PFBC01 uptake and systemic tumor burden.

### Safety analysis

For safety analysis of ^68^Ga-PFBC01 PET/CT administration, participants were monitored for adverse events during and within 2 hours after injection. To assess delayed adverse events, an additional on-site visit at 7 days plus or minus 1 day and a telephone follow-up at 30 days plus or minus 3 days after ^68^Ga-PFBC01 PET/CT were conducted. Heart rate, blood pressure, ECG, and physical examination were assessed during the on-site visit.

### Sample size

For the primary endpoint (sensitivity), sample size was calculated using a 1-sample proportion superiority design with normal approximation. We tested H0: *P* = 0.70 versus H1: *P* = 0.90. Using a 2-sided α = 0.05 and power 90%, the calculation yields *n* ≈ 41.1, which was rounded up to 42 participants. Allowing for an anticipated 15% dropout rate results in a planned recruitment target of 50 participants (42/(1–0.15) ≈ 49.4, rounded up).

### Image interpretation blinding

Both ^68^Ga-PFBC01 PET/CT and ^18^F-FDG PET/CT scans were independently reviewed by 3 nuclear medicine physicians with over 3 years of experience. Image datasets were anonymized before reviewing. Readers were provided with attenuation-corrected PET images and the corresponding anatomical CT data. Except for basic information (age and uptake time), all other clinical data were masked. Discrepancies were resolved by majority consensus.

### Definition of overall burden in PET/CT

Following previous protocol (44), an artificial intelligence–based algorithm automatically segmented skeletal structures from CT scans into 3 anatomical regions: thoracic, spinal, and pelvic bones, which represent the most frequently involved sites in MM (Supplemental Figure 2A). Segmentation and quantification were performed using a standardized automated workflow to minimize inter-observer variability and improve reproducibility across patients. All segmentations underwent visual quality review by experienced investigators, with manual correction performed only when necessary. For each anatomical region, the mean standardized uptake value (SUVmean) was calculated to quantify regional disease burden while reducing bias related to differences in skeletal volume among individuals. The sum of thoracic, spinal, and pelvic SUVmean values was defined as the overall burden (Supplemental Figure 2B). Unlike metabolic tumor volume (MTV) or total lesion glycolysis (TLG), which primarily depend on lesion threshold delineation and may be influenced by heterogeneous lesion morphology, this regional SUVmean–based approach was designed to provide a robust and anatomically standardized assessment of whole-body disease activity.

### Clinical decision making and response evaluation

Clinical decisions were independently made by 3 hematologists, each with more than 3 years of clinical experience. Initial treatment plans were formulated based on the patient’s medical history, laboratory findings, bone marrow biopsy results and ^18^F-FDG PET/CT findings. After review of the ^68^Ga-PFBC01 PET/CT findings, treatment strategies were subsequently revised as appropriate. Any discrepancies in clinical judgment were resolved by majority consensus. These treatment-guidance analyses were exploratory and intended to evaluate the potential clinical utility of ^68^Ga-PFBC01 PET/CT rather than establish predefined therapeutic algorithms.

Treatment response was evaluated according to International Myeloma Working Group consensus criteria for response (45). The response criteria in ^18^F-FDG PET/CT were established according to the 2021 Deauville Criteria (4), including complete metabolic response (CMR) and partial metabolic response (PMR).

### Pathological examination

Formalin-fixed, paraffin-embedded biopsy specimens were subjected to immunohistochemical staining for BCMA, CD138, and Vs38c following standard laboratory protocols, as previously described (46).

### Protocol and statistical analysis plan

Protocol and statistical analysis plan could be found in the trail protocol file.

### Statistics

Primary endpoints were analyzed in the intention-to-treat population, defined as all enrolled participants who received ^68^Ga-PFBC01 PET/CT. All participants who received ^68^Ga -PFBC01 were included in the safety population. Patient-based and region-based diagnostic performance (primary endpoints) were calculated as preplanned. The corresponding 95% confidence interval was calculated using the Clopper–Pearson exact binomial method. Patient-based and region-based analyses were interpreted independently. Interreader agreement for both modalities was reported at the per-patient and per-region levels using Fleiss’ κ and interpreted according to Landis and Koch criteria (47). Descriptive data for safety outcomes and adverse events were presented. For secondary outcomes, correlations were assessed using Spearman’s rank correlation coefficient, and differences between groups were analyzed with the Wilcoxon rank-sum test. All analyses were performed using R software (version 4.4.1). A 2-sided *P* value < 0.05 was considered statistically significant. The final analysis was performed on October 10, 2025.

### Study approval

All mouse and nonhuman primate studies were approved by the Ethics Committee of Peking University First Hospital (No. 2024171) and were conducted in accordance with relevant institutional guidelines for the care and use of laboratory animals. This study involving human participants was conducted in accordance with the ethical standards of the institutional and national research committee and with the 1964 Declaration of Helsinki and its later amendments. The study protocol was reviewed and approved by the Ethics Committee of Peking University First Hospital (No. 2024171 and Approval No. 2024600-002) and registered on ClinicalTrials.gov (NCT06717113). All participants provided written informed consent prior to enrollment. All human biological samples were collected under institutional biosafety and ethical regulations and were used exclusively for diagnostic and research purposes within the approved protocol. The privacy and confidentiality of all participants were strictly maintained.

The study protocol was approved by the Ethics Committee of Peking University First Hospital (Approval No. 2024600-002) and registered on ClinicalTrials.gov (NCT06717113; https://clinicaltrials.gov/study/NCT06717113?cond=BCMA%20PET&rank=1).

### Data availability

Supporting data values for all figures are provided in the Supporting Data Values file.

## Author contributions

TG, ZC, and BT coordinated patient recruitment, clinical data collection, and imaging procedures. BT, HL, ZL, QW, YZ, YS, MD, TY, and YQ were involved in patient management, clinical evaluation, data collection, and interpretation of clinical and hematological data. Imaging acquisition and interpretation were performed by TG, ZC, QY, LS, and SW. TW was responsible for tracer synthesis and radiochemical preparation, and QY and Yimeng Du contributed to tracer-related biological validation. WW provided pathological expertise and performed pathological analyses supporting the imaging-pathology correlation. Statistical analyses were performed by TG. TG had unrestricted access to the full dataset and verified the statistical results. LK and Yujun Dong also had unrestricted access to all study data and oversaw the integrity of the data and the statistical analyses. TG prepared the first draft of the manuscript. XX, Yujun Dong, and LK critically reviewed, revised, and edited the manuscript for important intellectual content. TG, ZC, BT, TW, and QY contributed equally to this work. The order of co-first authors was determined by the extent of their contributions and mutual agreement among the authors. All authors confirm that they agreed to submit the manuscript for publication, have read and approved the final version, and take full responsibility for the accuracy and completeness of the data, the fidelity of the study to the registered protocol, and the validity of the statistical analyses.

## Conflict of interest

The authors have declared that no conflict of interest exists.

## Funding support

- National Natural Science Foundation of China (82472018 to LK, 82402320 to TW).
- Beijing Nova Program (20240484725 to LK).
- National High Level Hospital Clinical Research Funding (Interdisciplinary Research Project of Peking University First Hospital, 2024IR07, Scientific and Technological Achievements Transformation Incubation Guidance Fund Project of Peking University First Hospital, 2025CX38, 2024CX18 to LK; Research Achievement Transformation Project of Peking University First Hospital, 2025ZH02 to ZC).
- Clinical Medicine Plus X - Young Scholars Project of Peking University, the Fundamental Research Funds for the Central Universities (PKU2026PKULCXQ038 to ZC).

## Supplementary Material

Supplemental data

Supplemental data 2

ICMJE disclosure forms

Unedited blot and gel images

Supporting data values

## Version 1 06/30/2026

In-Press Preview

## Version 2 09/15/2026

Electronic publication

## Version 3 10/16/2026

Added English clinical study protocol sd2
