# Supplementary material for: A single-arm prospective phase I trial of 68Ga-PFBC01 PET/CT for multiple myeloma B cell maturation antigen imaging
Source: J Clin Invest. 2026 Jun 30;136(18):e207391. doi: 10.1172/JCI207391 (PMC13574143; doi:10.1172/JCI207391)
Supplement: Supplementary file 1 [file jci-136-207391-s008.pdf]

Supplementary data

A single-arm prospective phase I trial of ^{68}Ga-PFBC01 PET/CT for multiple myeloma B cell maturation antigen imaging

Tingfei Gu[1 #], Zhao Chen[1 #], Bo Tang [2 #], Tianyao Wang[1 #], Qi Yang[1 #], Huihui Liu [2], Zeyin Liang [2], Qian Wang [2], Yang Zhang [2], Yuhua Sun [2], Mingyi Di [2], Tingting Yuan [1], Yongkang Qiu [1], Yimeng Du [3], Lele Song [1], Shengnan Wu [1], Wei Wang [4], Xiaojie Xu [3 *], Yujun Dong [2 *], Lei Kang [1 *]

[1] Department of Nuclear Medicine, Peking University First Hospital, Beijing 100034, China.

[2] Department of Hematology, Peking University First Hospital, Beijing 100034, China.

[3] Department of Genetic Engineering, Beijing Institute of Biotechnology, Beijing, 100071, China

[4] Department of Pathology, Peking University First Hospital, Beijing 100034, China.

Supplementary data 1
A single-arm prospective phase I trial of ^{68}Ga-PFBC01 PET/CT for multiple myeloma B cell maturation antigen imaging 1
Methods for studies performed in primates and mice 3
Graphical abstract 5
Figure S1 In-vitro stability of ^{68}Ga-PFBC01 in (A) PBS and (B) FBS from 15 minutes to 4 hours, as assessed by radio-TLC. 6
Figure S2 Schematic illustration of overall burden calculation on PET/CT 7
Figure S3 Elevated blood-pool uptake on ^{68}Ga-PFBC01 PET/CT and its clinicopathologic correlations 9
Figure S4 Whole-blood pharmacokinetic modeling of ^{68}Ga-PFBC01 in healthy non-human primates. 11
Figure S5 Association between renal function and kidney uptake of ^{68}Ga-PFBC01 PET/CT 12
Table S1 Preliminary Dosimetry Estimates of ^{68}Ga-PFBC01 in Non-human Primates . 14
Table S2 Inter-scan Time Differences Between ^{68}Ga-PFBC01 and ^{18}F-FDG PET Studies 15
Table S3 Basic characteristics of patients. 16
Table S4 Possibly related adverse events 17
Reference .... **错误！未定义书签。**

Methods for studies performed in primates and mice

Cell Lines

Human multiple myeloma cell lines H929, MM.1S, and human chronic myelogenous leukemia cell line K562, were obtained from the American Type Culture Collection (ATCC) and cultured in RPMI-1640 medium supplemented with 10% FBS and 1% penicillin–streptomycin at 37 °C in 5% CO_2 .

Animal Models

Female NCG (NOD/ShiLtJGpt-Prkdcem26Cd52Il2rgem26Cd22/Gpt) mice (4–6 weeks old) were purchased from GemPharmatech Co., Ltd. (Jiangsu, China). All animal studies were approved by the Animal Care and Use Committee of Peking University First Hospital (No. 2022018) and conducted in accordance with institutional guidelines. For subcutaneous tumor models, 5×10^6 H929 or K562 cells in 100 μL sterile PBS were implanted into the right armpit. Imaging and biodistribution studies were performed when tumor volumes reached 200–500 mm^3. An orthotopic MM.1S model was also established for imaging evaluation.

For non-human primate imaging, three healthy adult female cynomolgus monkeys (*Macaca fascicularis*, ~5 years old, 3-4 kg) were used. The animals were housed at Beijing Jinmuyang Laboratory Animal Breeding Co., Ltd. under controlled conditions, and all procedures were approved by the Laboratory Animal Ethics Committee of Peking University First Hospital (No. 2024171).

Flow Cytometry

To assess cell surface BCMA expression, 1×10^6 tumor cells were incubated with PFBC01 (25 μg/mL) for 30 min on ice. After washing, cells were stained with PE-conjugated anti-His tag antibody (1:200 dilution, clone / catalog number if available) for 30 min on ice. Cells stained with the PE-conjugated anti-His tag antibody alone served as the negative control. Following washing, cells were analyzed on a FACSCalibur flow cytometer and data were processed using FlowJo software.

In Vitro Cellular Uptake and Blocking Assays

Approximately 740 kBq of ^{68}Ga-PFBC01 was incubated with 1×10^6 cells in 200 μL culture medium for 30 min at 37 °C. Cells were washed three times with ice-cold PBS and radioactivity was

measured using an automated gamma counter. Blocking experiments were performed by co-incubating cells with 2×10^{-6} M unlabeled PFBC01 to confirm BCMA-specific binding. All uptake studies were performed in quadruplicate and repeated three times.

Small-Animal PET/CT Imaging

Tumor-bearing mice were intravenously injected with 11.1 MBq of ^{68}Ga-PFBC01 in 100 μL PBS via the tail vein. PET/CT scans were acquired at 1 h post-injection using a small-animal PET/CT scanner (Novel Medical). For blocking studies, mice were pre-injected with 30 μM unlabeled PFBC01 10 min prior to tracer administration. Regions of interest (ROIs) were drawn over major organs and tumors for quantitative analysis.

Ex Vivo Biodistribution

Mice were euthanized 1 h post-injection. Major organs and tissues (tumor, blood, heart, lung, liver, spleen, kidney, stomach, intestine, bladder, muscle, bone, and brain) were dissected, weighed, and counted for radioactivity using a gamma counter. Results were expressed as percentage of injected dose per gram of tissue (%ID/g).

PET/CT Imaging in Non-Human Primates

Dynamic PET/CT imaging was performed in healthy cynomolgus monkeys following intravenous injection of ^{68}Ga-PFBC01. PET images were acquired and reconstructed with CT-based attenuation correction. Quantitative ROI analysis was performed to evaluate tracer biodistribution and clearance kinetics.

Histopathology and Immunohistochemistry

Tumor xenografts were excised, fixed, sectioned, and stained with H&E. BCMA expression in tumor tissues was confirmed by immunohistochemistry (IHC) using anti-BCMA antibody. Histological evaluation was performed to confirm tumor phenotype and BCMA expression levels.

Therapy Response Monitoring

H929 tumor-bearing mice were treated with the DVD regimen (daratumumab, bortezomib, and dexamethasone) according to established protocols. PET imaging was performed before treatment and on days 5 and 10 post-treatment to monitor therapeutic response, with quantitative analysis of tumor tracer uptake.

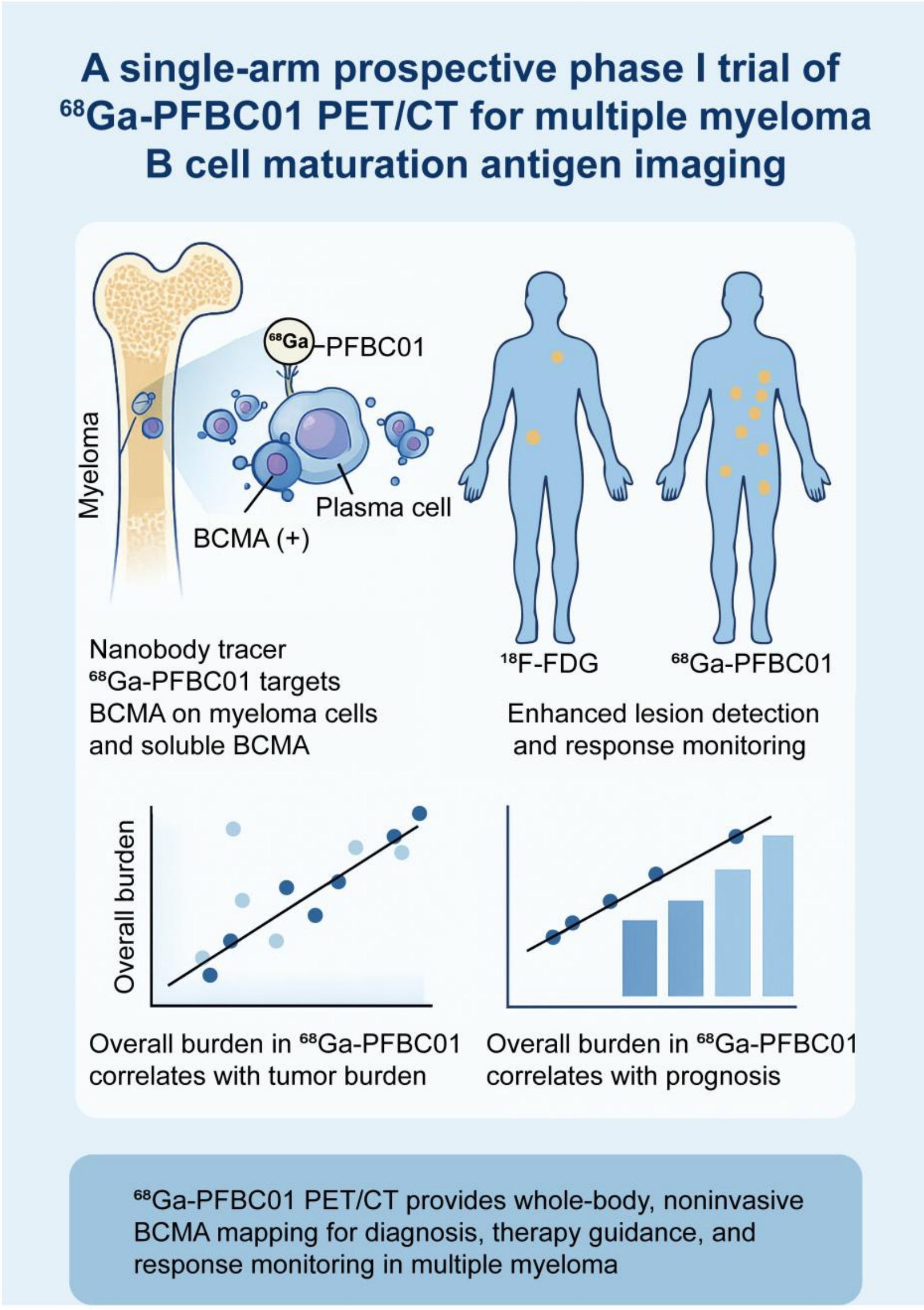

Graphical abstract

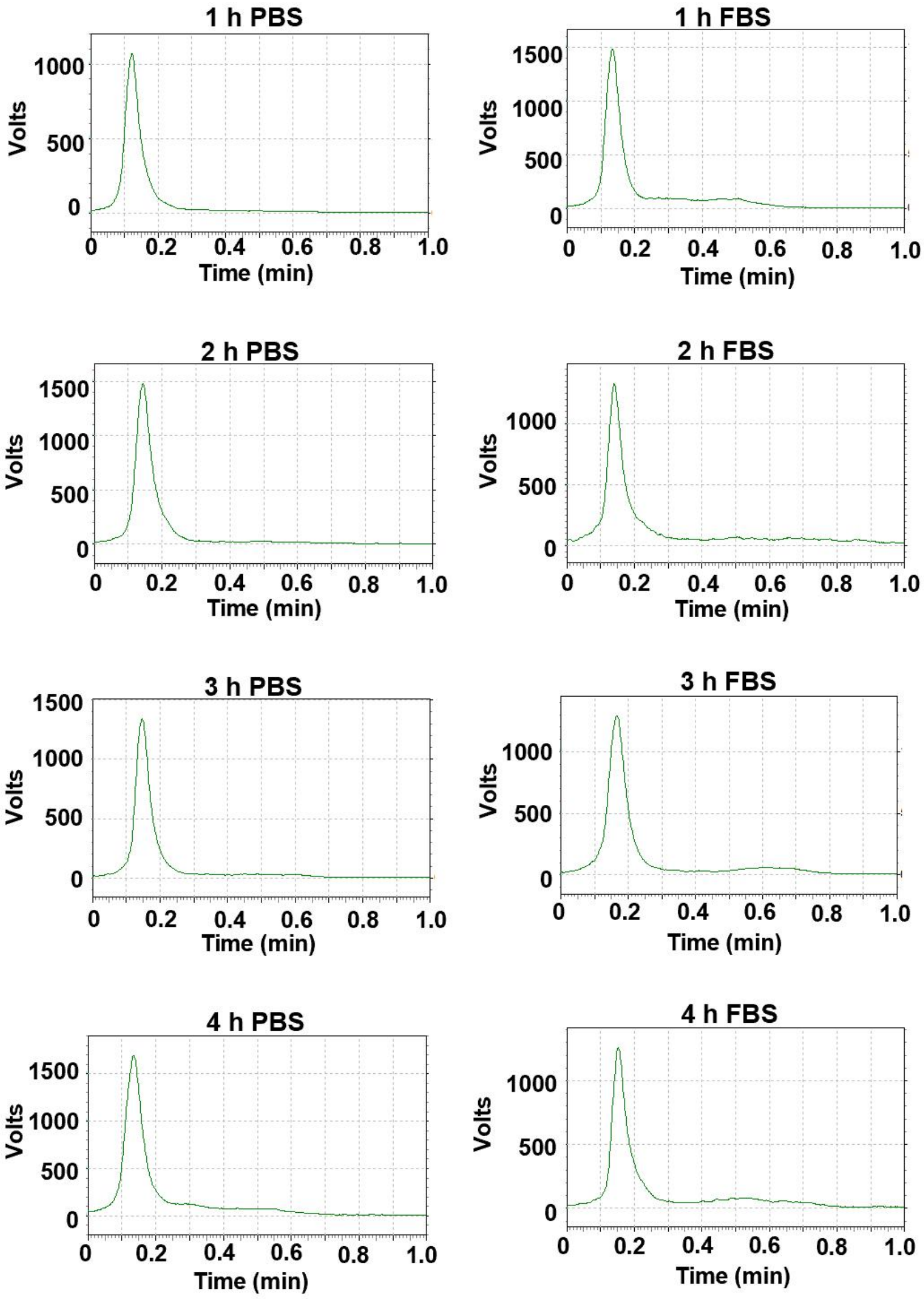

Figure S1 In-vitro stability of ^{68}Ga-PFBC01 in (A) PBS and (B) FBS from 15 minutes to 4 hours, as assessed by radio-TLC.

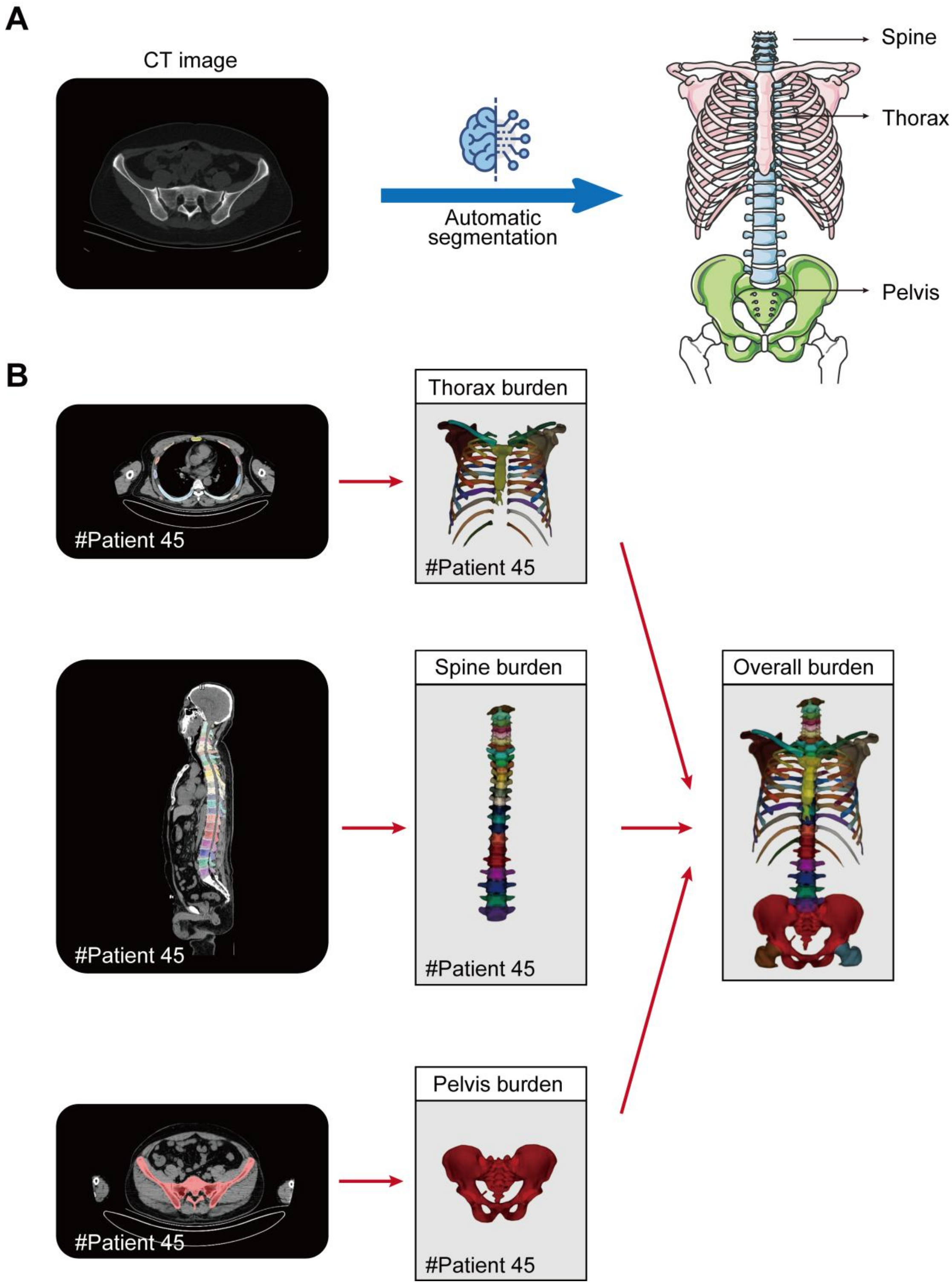

Figure S2 Schematic illustration of overall burden calculation on PET/CT

A. Artificial intelligence–based segmentation of skeletal structures on CT images for automated quantification of metabolic activity. The algorithm identifies the entire bony framework and calculates the mean standardized uptake value (SUVmean) for each region, enabling objective, reproducible PET-based assessment of skeletal involvement.

B. Example of automated skeletal segmentation and burden quantification in Patient No.45. The algorithm independently identifies the thoracic bones, spine, and pelvis to calculate

corresponding SUVmean values, defined as thorax burden, spine burden, and pelvis burden, respectively. The overall burden is then computed as the sum of thorax, spine, and pelvis burden values.

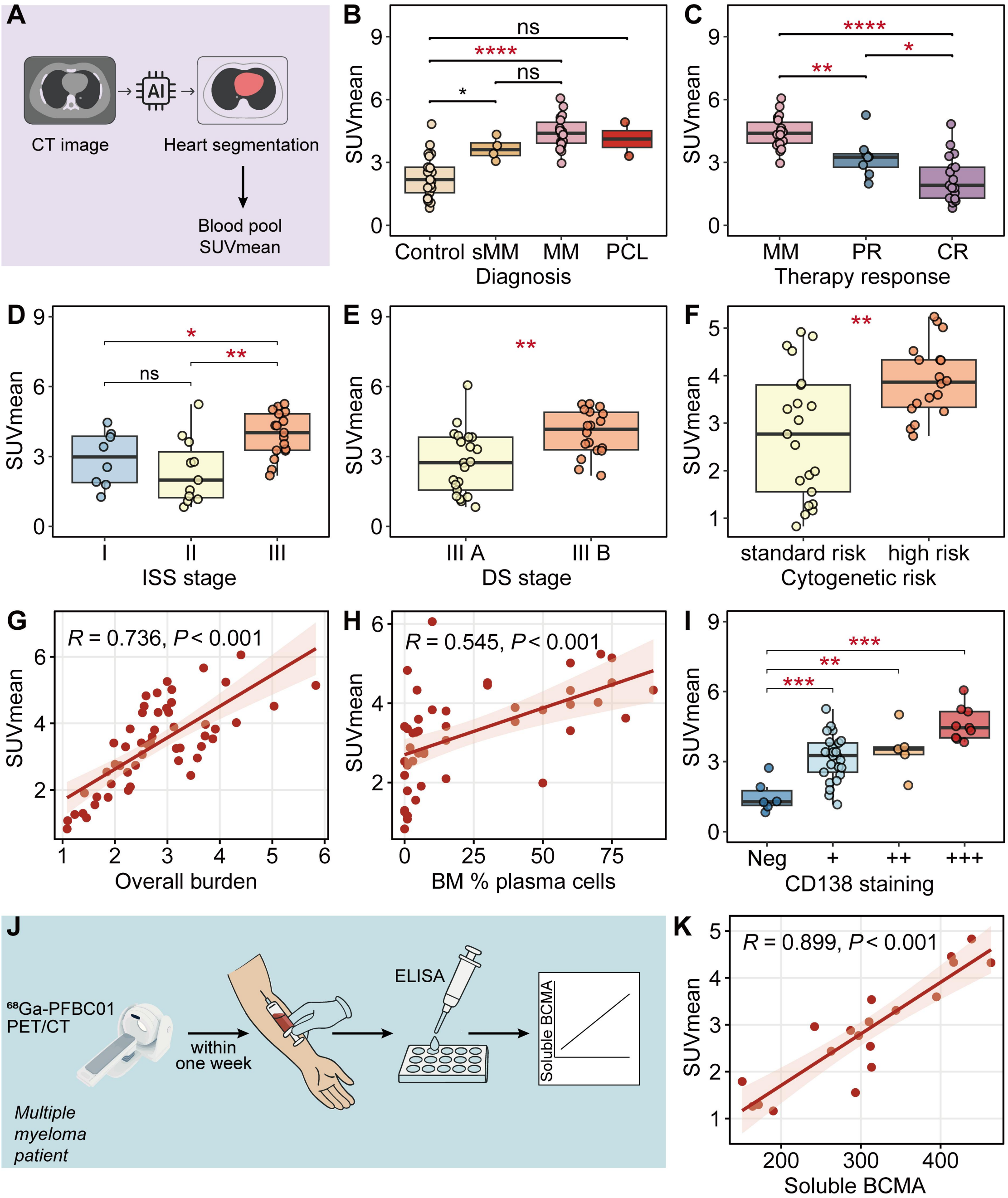

Figure S3 Elevated blood-pool uptake on ^{68}Ga-PFBC01 PET/CT and its clinicopathologic correlations

A. Schematic illustration of artificial intelligence–assisted segmentation on CT images for automated quantification of cardiac blood-pool uptake
B. Comparison of blood-pool uptake among plasma cell disorders: control patients, smoldering multiple myeloma (sMM), MM, and plasma cell leukemia (PCL).
C. Blood-pool uptake across treatment response categories: multiple myeloma (MM, untreated), partial response (PR), and complete response (CR).
D. Prognostic evaluation of blood-pool uptake according to International Staging System (ISS) stage.
E. Prognostic evaluation of blood-pool uptake across Durie–Salmon (DS) stages.

F. Prognostic evaluation of blood-pool uptake based on cytogenetic risk.

G. Correlation between blood-pool uptake and overall burden on ^{68}Ga-PFBC01 PET/CT.

H. Correlation between blood-pool uptake and bone-marrow plasma-cell percentage.

I. Blood-pool uptake on ^{68}Ga-PFBC01 PET/CT in patients with pathologically confirmed multiple myeloma (MM) across different CD138 immunohistochemistry (IHC) scores.

J. Within one week of ^{68}Ga-PFBC01 PET/CT, soluble BCMA levels in patient plasma using enzyme-linked immunosorbent assay (ELISA) were measured.

K. Correlation between blood-pool uptake and serum soluble BCMA concentration.

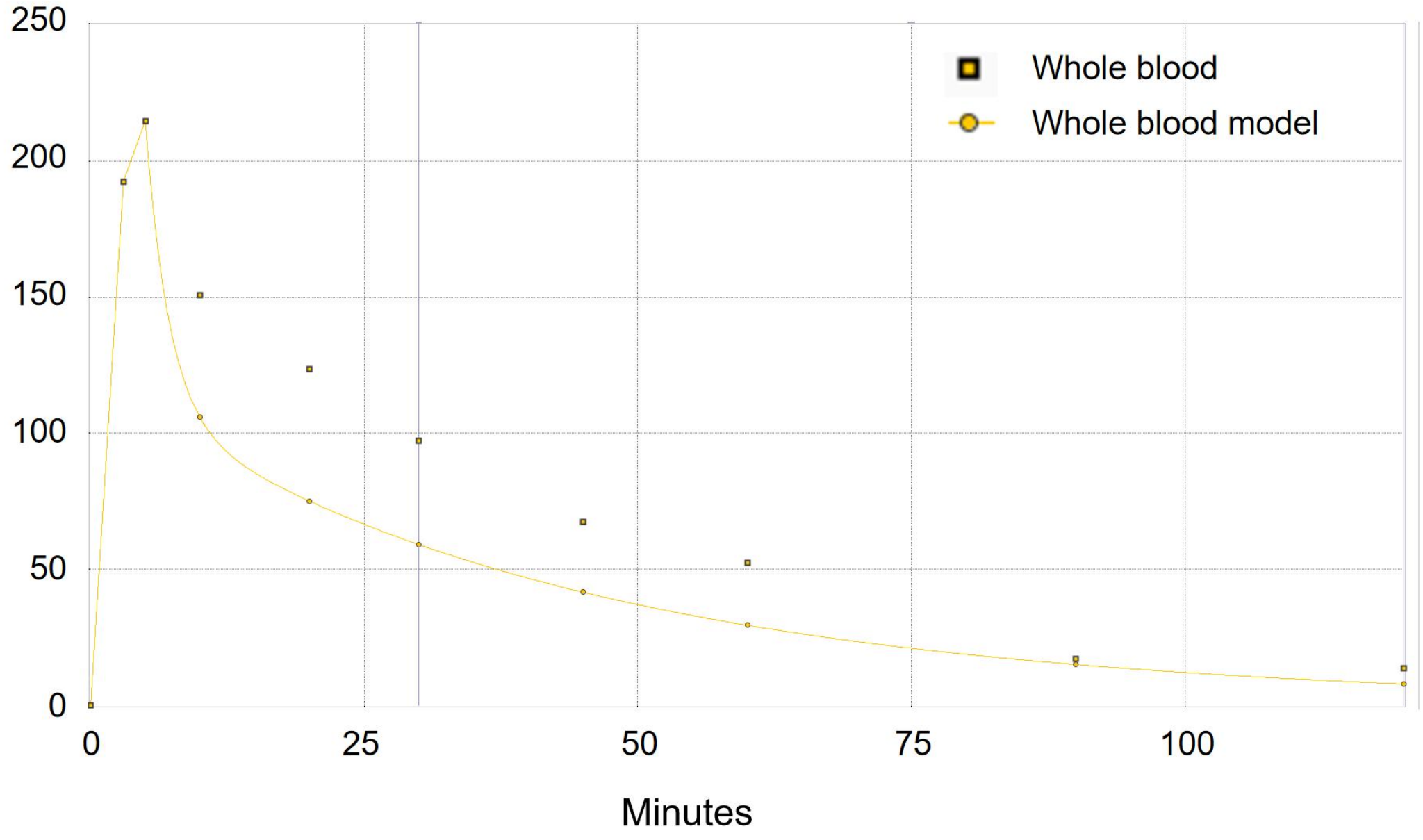

Figure S4 Whole-blood pharmacokinetic modeling of ^{68}Ga-PFBC01 in healthy non-human primates.

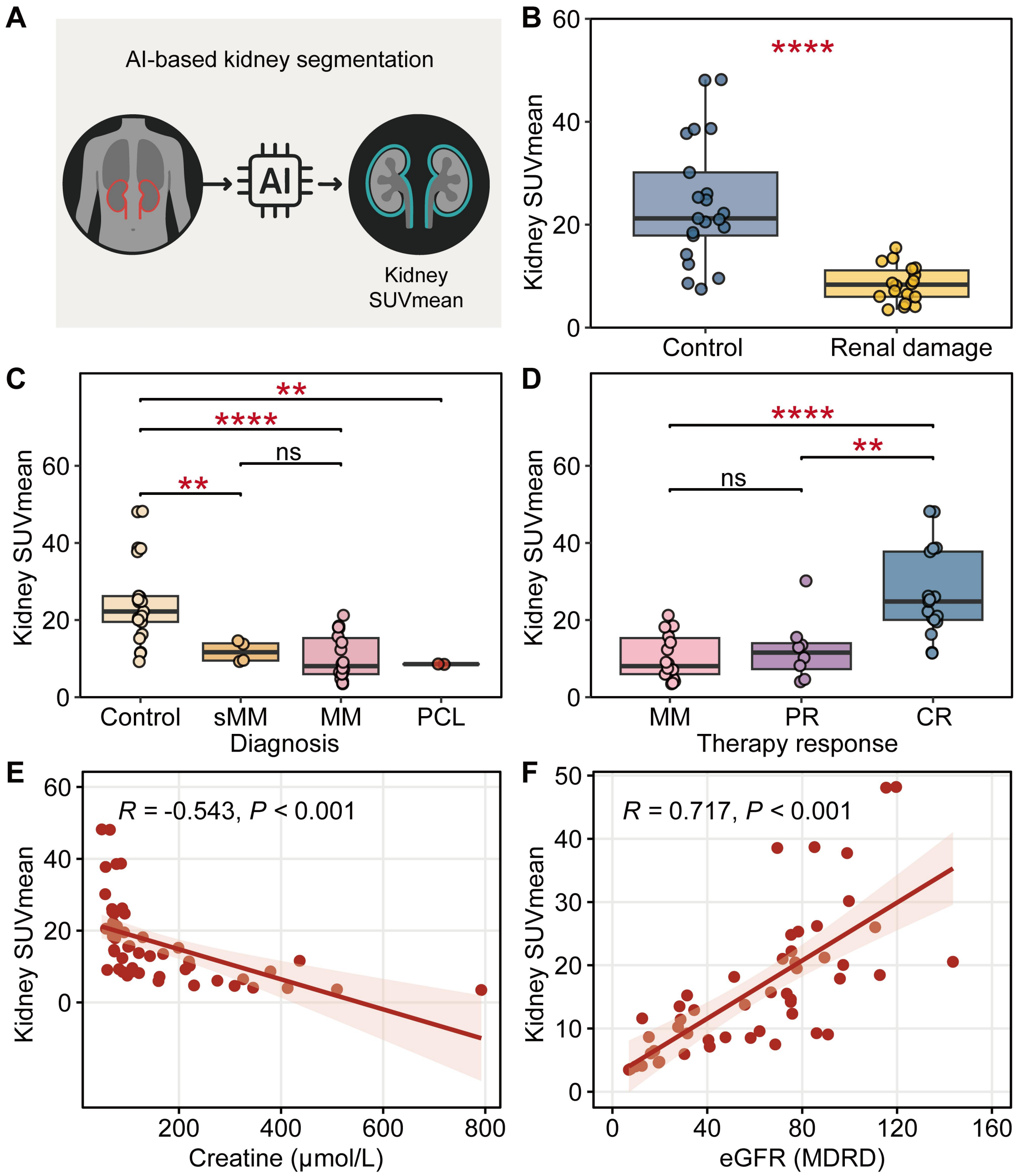

Figure S5 Association between renal function and kidney uptake of ^{68}Ga-PFBC01 PET/CT

A. Schematic illustration of the artificial intelligence–based kidney segmentation workflow for automated quantification of kidney SUVmean.

B. Comparison of kidney SUVmean between participants with normal renal function (control) and renal impairment.

C. Kidney SUVmean across different plasma cell disorder subgroups, including smoldering multiple myeloma (sMM), multiple myeloma (MM), and plasma cell leukemia (PCL).

D. Kidney SUVmean according to treatment response categories, including active MM, partial

response (PR), and complete response (CR).

E. Correlation between kidney SUVmean and serum creatinine levels.

F. Correlation between kidney SUVmean and estimated glomerular filtration rate (eGFR, MDRD equation).

Table S1 Preliminary Dosimetry Estimates of ^{68}Ga-PFBC01 in Non-human Primates

Organ	Sample 1	Sample 2	Sample 3
Adrenals	4.41E-04	3.68E-03	9.24E-04
Brain	5.81E-06	6.42E-06	4.58E-06
Breasts	1.11E-04	9.18E-05	8.12E-05
Esophagus	4.71E-04	3.85E-04	3.48E-04
Eyes	7.30E-06	7.00E-06	5.58E-06
Gallbladder Contents	1.95E-04	7.75E-04	2.40E-04
Left colon	2.58E-04	3.85E-04	2.39E-04
Small Intestine	6.12E-05	2.71E-04	8.79E-05
Stomach Wall	1.90E-04	5.51E-04	2.08E-04
Right colon	7.45E-05	2.75E-04	8.82E-05
Rectum	9.32E-06	4.79E-05	1.53E-05
Heart Wall	8.67E-03	3.01E-03	6.20E-03
Kidneys	2.92E-03	3.52E-02	1.07E-02
Liver	2.20E-03	3.74E-03	1.02E-03
Lungs	3.39E-04	1.06E-03	4.08E-04
Ovaries	1.58E-05	8.12E-05	2.57E-05
Pancreas	2.26E-04	6.42E-04	2.14E-04
Salivary Glands	1.63E-05	1.60E-05	1.26E-05
Red Marrow	8.79E-05	1.81E-04	8.59E-05
Osteogenic Cells	4.93E-05	1.05E-04	4.85E-05
Spleen	6.17E-04	1.68E-02	2.99E-03
Thymus	4.09E-04	2.49E-04	3.05E-04
Thyroid	8.15E-05	7.61E-05	6.37E-05
Urinary Bladder Wall	7.83E-06	3.38E-05	1.11E-05
Uterus	1.35E-05	6.59E-05	2.12E-05
Total Body	2.16E-04	4.57E-04	2.06E-04

Table S2 Inter-scan Time Differences Between ^{68}Ga-PFBC01 and ^{18}F-FDG PET Studies

Time Difference (days)	Number of Subjects
1	15
2	5
3	7
4	6
5	4
7	1
9	1
12	1

Table S3 Basic characteristics of patients.

	^{68}Ga-PFBC01 PET (n = 50) *
Age, median (IQR)	62 (55-66)
Sex	
Female	23 (46%)
Male	27 (54%)
Race and ethnicity	
Asian	50 (100%)
Diagnosis	
Smoldering multiple myeloma (sMM)	4 (8%)
Multiple myeloma (MM)	18 (36%)
Non multiple myeloma (control)	4 (8%)
Plasma cell leukemia (PCL)	2 (4%)
Complete response (CR)	16 (32%)
Partial response (PR)	6 (12%)

*: A total of 50 patients were enrolled in the study. Among them, one patient underwent two examinations, and another patient underwent three examinations.

Table S4 Possibly related adverse events

	Grades 1–2	Grades 3	Grades 4	Grades 5
Any	2 (4%)	0	0	0
Nausea	1 (2%)	0	0	0
Dizziness	1 (2%)	0	0	0

Possibly related adverse events documented within 7 days after ^{68}Ga-PFBC01 PET/CT in two (4%) of 50 participants. Graded according to Common Terminology Criteria for Adverse Events (version 5.0) and classified as possibly attributable to ^{68}Ga-PFBC01 PET/CT.
