# Supplementary material for: A single-arm prospective phase I trial of 68Ga-PFBC01 PET/CT for multiple myeloma B cell maturation antigen imaging
Source: J Clin Invest. 2026 Jun 30;136(18):e207391. doi: 10.1172/JCI207391 (PMC13574143; doi:10.1172/JCI207391)
Supplement: Supplementary file 2 [file jci-136-207391-s009.pdf]

Project Name: ^{68}Ga-BCMA probe in multiple myeloma

PET imaging studies

Clinical study protocol

Project/Scheme Number: BCMA001

Version number: V1.0

Version date: 202410.08

Project source: self-raised

Sponsor: Peking University First Hospital

Project Leader: Lei Kang

Department of the unit: Department of Nuclear Medicine

Contact number: 010-83575252

Project implementation time: November 2024 to June 2027

1. Introduction/Background/Principles of Research

Multiple myeloma (MM) is an age-related malignant bone marrow plasma cell proliferative disorder with monoclonal immunoglobulin or light chain (M-protein) overproduction. MM is more common in the elderly group, with an insidious onset, and most of them are in the middle and late stages when diagnosed. In the United States, MM exceeded 30,000 cases and 13,000 deaths in 2016[1]. With the aging of our country's population, the incidence of MM continues to rise, surpassing that of acute leukemia. The diagnosis of MM mainly relies on the increase in clonal plasma cells in bone marrow biopsy, but because bone marrow aspiration is an invasive procedure and the puncture site is improperly selected, false negatives may occur[2]. Compared with traditional imaging modalities, nuclear medicine imaging technology can non-invasively and accurately reflect tissue function and metabolic changes due to its high sensitivity and ability to bind with specific probes. Therefore, the development of new nuclear medicine molecular probes for the diagnosis and treatment of MM is of great clinical significance.

B-cell maturation antigen (BCMA) is a member of the tumor necrosis factor superfamily, encoded by the TNFRSF17 gene on chromosome 16, and belongs to type III transmembrane glycoprotein[3]. In MM, BCMA binds to its ligand B cell activation factor (BAFF) and proliferation-inducing ligand (APRIL), activating multiple growth signaling pathways, including NF-κB, MAPK, and PI3K-Akt, to promote cell proliferation and anti-apoptotic responses[4]. BCMA also regulates PD-L1, TGF-β, and IL-10 upwardsA variety of immunosuppressive molecules promote immune escape in MM cells. A large number of studies have confirmed the highly selective expression of BCMA on the surface of almost all MM cell lines (80~100%)[5], supporting it as an ideal target site for MM diagnosis and treatment[6, 7].

In recent years, BCMA-targeted therapies such as CAR-T cell therapy, bispecific antibodies (BsAbs), and antibody-drug conjugates (ADCs) have shown promising therapeutic potential[8] [9] [10] [3, 11] [12]. These new therapies have shown good efficacy in patients with relapsed and refractory MM, but they are also accompanied by certain

toxic reactions, including cytokine release syndrome and off-target effects[3, 6, 13]. In addition, some patients did not benefit significantly, suggesting the need to develop more effective monitoring methods to assess BCMA expression and treatment response in a noninvasive manner.

Nuclear medicine technology can detect the vital distribution of target molecules in vivo by labeling specific molecular probes with radionuclides using single-photon computed tomography (SPECT) or positron emission tomography (PET/CT). The commonly used imaging agent 18F-FDG is widely used in the diagnosis, staging, and efficacy monitoring of malignant tumors.However, there are certain limitations in the detection of MM, due to the low expression of glucose transporter (GLUT1) and hexokinase-2 (HK-2) in MM cells^{18}F-FDG is difficult to detect some intramedullary lesions[14]. In contrast, immunoPET can combine the high specific binding ability of antibodies with the high sensitivity and quantitative analysis advantages of PET imaging.Target expression and distribution can be displayed at the living level. This detection has great potential for the detection of specific targets in multiple myeloma.

Different types of antibody drug fragments have their own advantages in tumor targeted diagnosis and treatment. Due to their large molecular weight, radiolabeled total antibodies usually need to bind to radionuclides with long half-lives, resulting in slow pharmacokinetics, slower blood clearance, and higher background signals, but it can achieve targeted endobeam therapy by specifically binding to tumor cells[15]. In contrast, peptide drug conjugates (PDCs) are suitable for use with diagnostic nuclides with short half-lives such as small molecular weight, excellent tumor penetration, and low immunogenicity^{18}F、^{68}Ga) with good clinical translation potential[16]. As the smallest structural antibody fragment, nanobodies (Nb) have high antigen affinity, tissue permeability, and faster clearance rates, and are commonly used for molecular imaging and therapeutic delivery. Antibody fragments are preferred candidates for immunoPET imaging agents by retaining the targeting properties of intact antibodies, with better tumor penetration, faster blood clearance, and higher signal-to-noise ratio[17].

At present, significant progress has been made in the research and development of targeted BCMA probes. For example, a team from the Department of Nuclear Medicine

at the First Affiliated Hospital of Jinan University recently developed a monoclonal antibody BCMAh230430 that targets BCMA, which is transmitted through 89Zr and^{177Lu}After labeling separately, its effectiveness in non-invasive monitoring of BCMA expression status and radioimmunotherapy in multiple myeloma (MM) subcutaneous tumors was demonstrated[18]. Wei et al[19]Two nanobodies targeting BCMA were designed for the detection of primary multiple myeloma models, and PET scintigraphy analysis showed that the diagnostic effects of these two tracers in the disseminated MM model were comparable and could effectively detect disseminated MM lesions. At the same time, the ^{68}Ga-labeled BCMA-targeted peptide BP1 developed by our team showed excellent tumor targeting and in vivo stability in preclinical studies, mainly aggregating in kidney, bladder and BCMA-positive H929 tumors[20].

Preclinical study of the ^{68}Ga-BCMA probe

^{68}Ga-BCMA probe preparation process:

1) Configure the BCMA precursor as 1 mg/mL of aqueous solution, mix 20 μL of the precursor with 195 μL of sodium acetate solution (1.0 M), set aside;

2) Leaching the germanium-gallium generator with 0.05 M hydrochloric acid, discarding the 1st mL, adding the remaining 3 mL to the reaction system in step 1, heating the reaction at 90oC for 10 minutes;

3) Pre-activate the Sep-Pak C18 purification column for backup, and the activation method is 5 mL ethanol, 5 mL water, and 10 mL air pass through C18 in turn.

4) At the end of the reaction, 2 mL of normal saline was added to the system, and the entire reaction system was purified by C18, and then the C18 column was washed with 0.7 mL of anhydrous ethanol and 7 mL of normal saline, and then passed through a 0.22 μm microporous filter membrane to obtain the final product for injection by the subject.

5) Take an appropriate amount of aseptically filtered product preparations for quality control inspection, and all items can be applied to subject imaging only after they are qualified.

^{68}Ga-BCMA preparation quality standard

According to the 2020 edition of the Pharmacopoeia of the People's Republic of

China, the quality standard for ^{68}Ga-BCMA preparations is formulated.

1) Appearance: This product should be a colorless clear solution, with no visible particulate matter. Inspect by visual inspection.

2) Identification: Take an appropriate amount of this product and measure it according to the half-life measurement method (Pharmacopoeia of the People's Republic of China, 2020 Edition, Part 4 General Principles 1401), and the half-life of the drug should comply with the regulations;

3) pH value: should be 5.0~8.0 (General Principles 0631 of the 2020 edition of the Pharmacopoeia of the People's Republic of China), measured with standard pH test strips.

4) Radiochemical purity: Take an appropriate amount of this product, determined by high performance liquid chromatography (General Principles 0512 of the 2020 edition of the Pharmacopoeia of the People's Republic of China), the chromatographic conditions are consistent with the differential detection, and the peak area of ^{68}Ga-BCMA radiochromatographic peak area is not less than 95% of the sum of all radiochromatographic peak areas .

5) Radioactive concentration: Take an appropriate amount of this product and measure it according to the radioactivity (concentration) determination method (General Rule 1401 of the 2020 edition of the Pharmacopoeia of the People's Republic of China), and the radioactivity concentration should not be less than 370 MBq/mL according to the time recorded on the label.

6) Bacterial endotoxin: Take an appropriate amount of medicine, check with bacterial endotoxin, dilute it with water at least 30 times, and then check it according to law ("Pharmacopoeia of the People's Republic of China" 2020 edition of the Four General Principles 1143), the amount of bacterial endotoxin per 1 ml of this product should be less than 15 EU.

7) Sterility: Take an appropriate amount of this product and check it in accordance with the law (General Principles 1101 of the Four Parts of the Pharmacopoeia of the People's Republic of China 2020 Edition), which should comply with the regulations

MicroPET scintigraphy of the ^{68}Ga-BCMA probe in tumor-bearing mice

NCG tumor-bearing mice (H929 and K562 tumors) were injected with a ^{68}Ga-BCMA probe and a negative control probe ^{68}Ga-NC (11 MBq, 200 µL) into the tail vein, respectively, followed by anesthesia under an isoflurane atmosphere (2% isoflurane - 30% oxygen/air), Scintigraphy studies were performed on PET/CT of Yongxin small animals. First, tumor-bearing mice were fixed on the scanning bed, and after injection, a dynamic whole body scan was performed for 1 h. As an inhibition test, tumor-bearing mice were injected with BCMA probe cold ligand (500 nmol/individual) at the same time for imaging studies. MicroPET imaging of tumor-bearing mice shows significant ^{68}Ga- BCMA probe uptake in kidneys and H929 tumors (BCMA+). In the inhibition test, the cold ligand BCMA probe significantly inhibited the uptake of the ^{68}Ga-BCMA probe in the tumor, indicating that the complex is highly specific for binding to BCMA proteins.

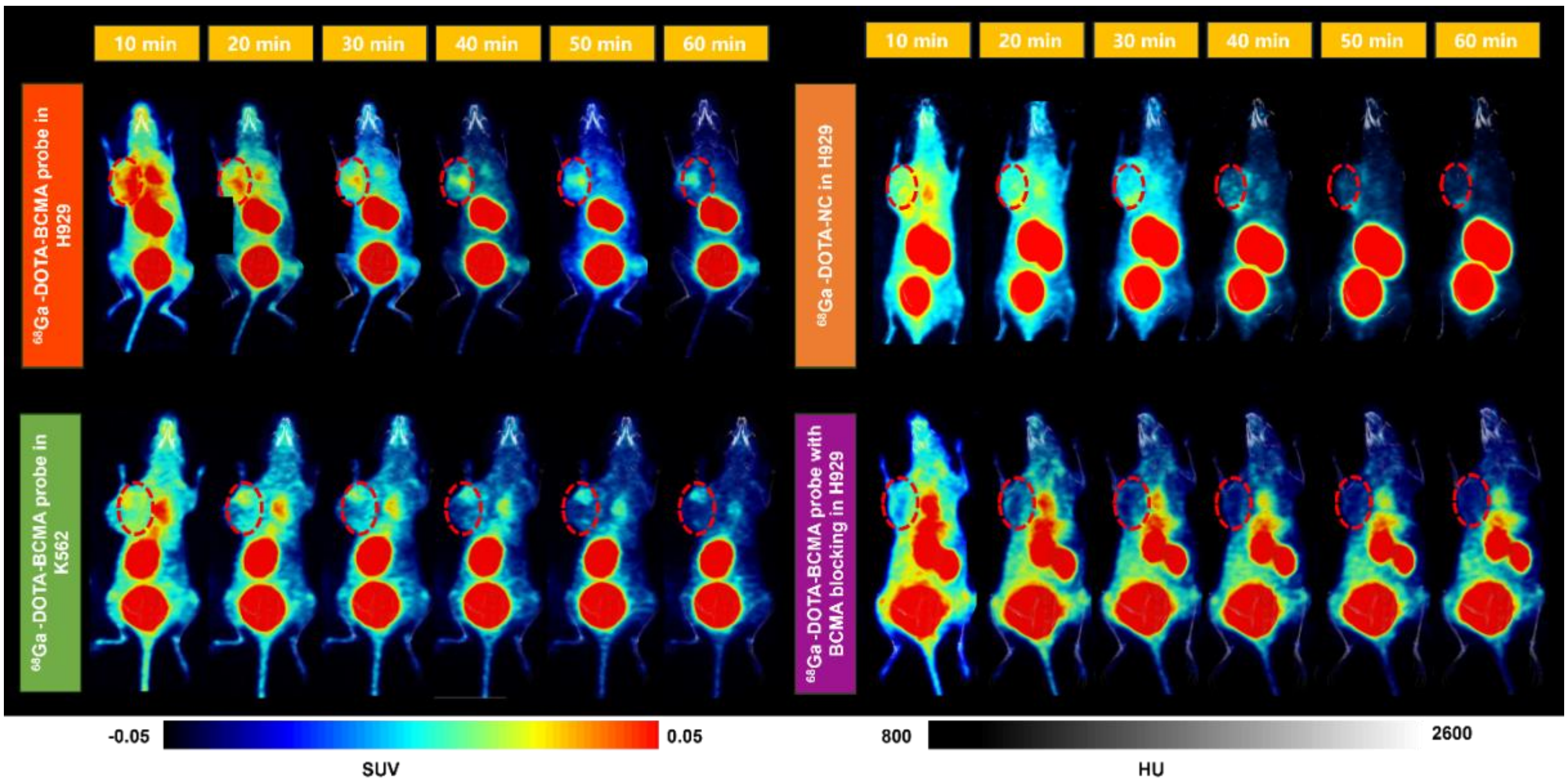

Figure 1 MicroPET whole-body imaging of tumor-bearing mice

Biodistribution study of ^{68}Ga-BCMA probes in tumor-bearing mice

NCG-bearing mice (H929-bearing tumors) were injected with a ^{68}Ga-BCMA probe (11 MBq, 200 µL) into their tails. Mice were euthanized 20 and 60 min after injection for biodistribution analysis. Various organs including blood, heart, lungs, liver, kidney, spleen, bladder, stomach, bones, muscles, small intestine, brain, and tumor were

collected and weighed and counted using a γ counter (Hidex). Radioactive uptake results are expressed as percent per gram of tissue (%ID/g, mean ± standard deviation). The results of the distribution of ^{68}Ga-BCMA probes in tumor-bearing organs are shown in Figure 2: During the animal distribution study, the vital signs and behavior of all mice did not change significantly.

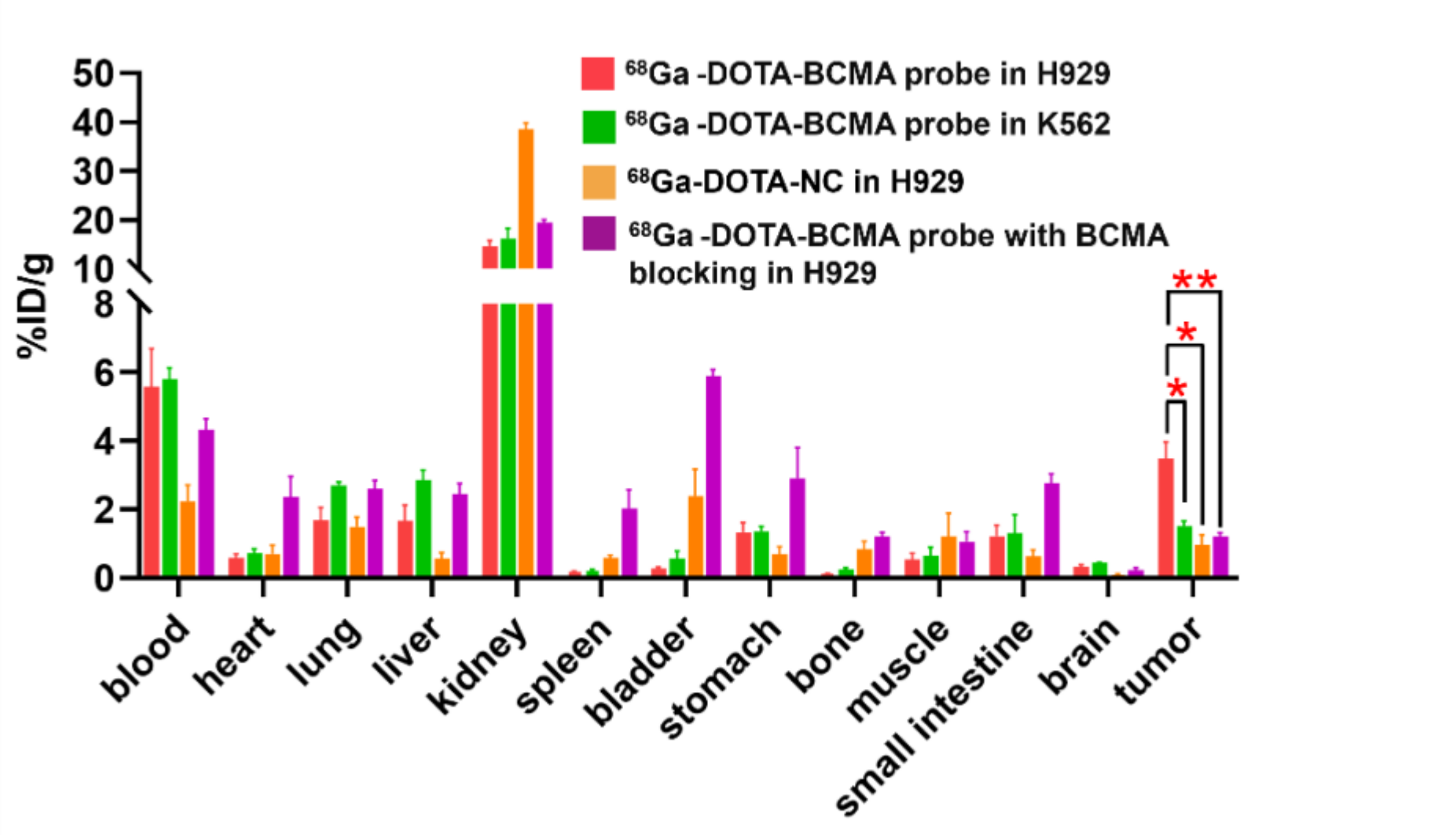

Fig. 2 Biodistribution in tumor-bearing mice

Through the above studies, targeted BCMA probes have shown a broad application prospect in MM molecular imaging. Future studies should further develop and optimize targeted BCMA probes to enhance their clinical translatability and verify their efficacy and safety in MM patients through more clinical trials.

2. Research purpose

This project uses BCMA protein as the targeting group of radiopharmaceuticals to explore the diagnostic efficacy of ^{68}Ga-BCMA probe in multiple myeloma, aiming to provide new methods and means for early diagnosis, accurate staging, recurrence judgment, treatment decision-making and prognosis judgment of multiple myeloma, and provide a scientific basis for the precise treatment of related diseases.

3. Research methods

3.1 The work completed so far and the conditions are met

The department is equipped with a GMP level radiopharmaceutical labeling room, and the test report is shown in Appendix 1, which can ensure the qualified preparation of ^{68}Ga-BCMA probe radiopharmaceuticals.

3.2 Research and design methods

This study is an interventional clinical study, and each patient will be randomized to receive a complete imaging of one of the ^{68}Ga-BCMA probe imaging agents, and the imaging will be randomized in the order of ^{18}F-FDG PET/CT scanning.

3.3 Research objects

3.3.1 Inclusion and exclusion criteria of the study population.

Inclusion Criteria:

1. Age≥ 18 years;
2. Suspected or pathologically confirmed diagnosis of multiple myeloma or related plasma cell disease;
3. ECOG performance status score ≤ 2 points;
4. Able to understand the content of the study and sign the written informed consent form.

Exclusion Criteria:

Pregnant or lactating women; Those with severe ECG abnormalities or unable to cooperate with PET/CT examination; Other conditions that the investigator deems unsuitable for participation in this study.

3.3.2 Sources and methods of research subject selection:

Research object source: Outpatients and inpatients of Peking University First Hospital.

Selection method of study subjects: Subjects who meet the conditions will be continuously screened for enrollment.

3.4 Research Steps

3.4.1 Collection of Information

According to the entry and placement criteria, patients who meet the conditions are screened. After the patients were enrolled, baseline examination information was collected, including blood routine, blood biochemistry, tumor markers, histopathological diagnosis results, imaging diagnosis results, etc.

3.4.2 Sample size and dosage used

This study was an exploratory study with 50 participants. Each subject will receive a single intravenous injection of ^{18}F-FDG and ^{68}Ga-BCMA probes, and PET/CT imaging will be performed within the specified time. The radiation dose used by the drug was about 0.08-0.1 mCi/kg

3.4.3 Reading diagnosis

All PET/CT images are jointly reviewed by at least three imaging and nuclear medicine physicians with many years of diagnostic experience and above the attending physician level, and a unified diagnostic opinion is given after consultation.

3.4.4 Follow-up

All adverse events will be systematically recorded and followed up during the study, and grading and causality judgment will be made according to the common adverse event grading criteria. This study has been approved by the Ethics Committee before implementation, and strictly follows the ethical principles of medical research and relevant regulatory requirements to ensure the rights and interests, privacy and data security of subjects.

Follow-up methods: Case follow-up was conducted through the Medix system of the Department of Nuclear Medicine, the PACS system of the Department of Medical Imaging, and the case system of Peking University First Hospital.

3.5 Statistical analysis of data

The statistical software uses R software (version 4.4.1). Standardized Uptake Value for each tumor lesion, SUV) were analyzed descriptively, including mean, standard deviation, coefficient of variation, minimum and maximum values. Statistically analyze the characteristics of SUVs over time. Plot the scatter distribution of SUVs in subjects. The SUV values of each tumor lesion in ^{18}F-FDG and ^{68}Ga-BCMA probe scintigraphies were compared, and the Pearson chi-square test was used for

comparison between the two groups, both with a difference of $P<0.05$ digits. The diagnostic sensitivity, specificity, negative predictive value, positive predictive value, and reader agreement of the two examination methods were calculated.

3.6 Data management

Centralized training and unified standards for data collection personnel.

All case data and imaging data are stored in a special database, and only the principal investigator is eligible to log in and read them. All case data will be anonymized before data analysis and transmission, and data transfer is limited to researchers in this project.

4. Subject protection

This study is an exploratory study, and the researcher will fully communicate the trial purpose, process, risks, possible benefits, etc. with the subjects before imaging, respect the subjects' personal wishes, and sign the informed consent form on this basis.

All case data were anonymized before data analysis. All case data and image data are stored in a special database, and only the principal investigator is qualified to log in and read them. All researchers are not allowed to disclose any information about any patient.

5. Quality control of research

Developer preparation stage: probe synthesis and marking personnel are trained uniformly, and the person in charge of each link is set up for quality control, and the probe is prepared in strict accordance with the test plan.

Imaging preparation stage: All researchers will be trained uniformly at the beginning of the study, and data collection will be carried out in strict accordance with the inclusion and exclusion criteria of the study subjects.

Imaging stage: drug injection by a senior nurse in nuclear medicine; Image acquisition by a senior nuclear medicine technician.

Image analysis stage: Joint reading and analysis by two attending nuclear medicine experts.

Data collation stage: centralized training and unified standards for data collection

personnel.

At the same time, the above teams include the informed consent group, the independent image acquisition group, the independent image analysis group, the independent data collation group, and the independent statistical analysis group.

6. Research schedule

2024.11-2027.02: Recruit cancer patients, perform PET/CT imaging evaluation of ^{18}F-FDG and ^{68}Ga-BCMA probes, and verify that the imaging agent ^{68}Ga-BCMA probe can safely and effectively identify lesions.

2027.02-2027.06: Data collation and summary and thesis writing, completion of research reports and paper publication.

7. Organization and management of research

This study is an interventional clinical study, the main investigators are from the Department of Nuclear Medicine and the Department of Hematology of Peking University First Hospital, and the project leader is unified arrangement, 2 people are probe preparation, 3 people are image acquisition and analysis, and 2 people are data analysis and paper writing. This study was completed with the cooperation and support of experts from the Department of Imaging, Hematology and other departments of our hospital.

Conflict of interest management plan: In this research plan, the patent R&D team is mainly responsible for the preparation and quality control of the reagent, and the patent R&D team does not participate in subject recruitment, image acquisition, image analysis, and will not participate in the statistical analysis of research data alone

8. References

[1] SIEGEL R L, MILLER K D, JEMAL A. Cancer statistics, 2018 [J]. CA Cancer J Clin, 2018, ^{68}(1): 7-30.

[2] KUMAR S K, RAJKUMAR S V, DISPENZIERI A, et al. Improved survival in multiple myeloma and the impact of novel therapies [J]. Blood, 2008, 111(5): 2516-20.

[3] YU B, JIANG T, LIU D. BCMA-targeted immunotherapy for multiple myeloma [J]. J Hematol Oncol, 2020, 13(1): 125.

[4] TAI Y T, ACHARYA C, AN G, et al. APRIL and BCMA promote human multiple myeloma growth and immunosuppression in the bone marrow microenvironment [J]. Blood, 2016,

127(25): 3225-36.

[5] ECKHERT E, HEWITT R, LIEDTKE M. B-cell maturation antigen directed monoclonal antibody therapies for multiple myeloma [J]. Immunotherapy, 2019, 11(9): 801-11.

[6] SHAH N, CHARI A, SCOTT E, et al. B-cell maturation antigen (BCMA) in multiple myeloma: rationale for targeting and current therapeutic approaches [J]. Leukemia, 2020, 34(4): 985-1005.

[7] WEI J, HAN X, BO J, et al. Target selection for CAR-T therapy [J]. J Hematol Oncol, 2019, 12(1): 62.

[8] TRUDEL S, LENDVAI N, POPAT R, et al. Targeting B-cell maturation antigen with GSK2857916 antibody-drug conjugate in relapsed or refractory multiple myeloma (BMA117159): a dose escalation and expansion phase 1 trial [J]. Lancet Oncol, 2018, 19(12): 1641-53.

[9] TAI Y T, MAYES P A, ACHARYA C, et al. Novel anti-B-cell maturation antigen antibody-drug conjugate (GSK2857916) selectively induces killing of multiple myeloma [J]. Blood, 2014, 123(20): 3128-38.

[10] USMANI S Z, GARFALL A L, VAN DE DONK N, et al. Teclistamab, a B-cell maturation antigen×CD3 bispecific antibody, in patients with relapsed or refractory multiple myeloma (MajesTEC-1): a multicentre, open-label, single-arm, phase 1 study [J]. Lancet, 2021, 398(10301): 665-74.

[11] DE NOVELLIS D, FONTANA R, GIUDICE V, et al. Innovative Anti-CD38 and Anti-BCMA Targeted Therapies in Multiple Myeloma: Mechanisms of Action and Resistance [J]. Int J Mol Sci, 2022, 24(1).

[12] RAJE N, BERDEJA J, LIN Y, et al. Anti-BCMA CAR T-Cell Therapy bb2121 in Relapsed or Refractory Multiple Myeloma [J]. N Engl J Med, 2019, 380(18): 1726-37.

[13] GUO R, LU W, ZHANG Y, et al. Targeting BCMA to Treat Multiple Myeloma: Updates From the 2021 ASH Annual Meeting [J]. Front Immunol, 2022, 13: 839097.

[14] KANG L, LI C C, YANG Q, et al. Cu-64-labeled daratumumab F(ab ')(2) fragment enables early visualization of CD38-positive lymphoma [J]. Eur J Nucl Med Mol I, 2022, 49(5): 1470-81.

[15] WU S, FAN K, YANG Q, et al. Smart nanoparticles and microbeads for interventional embolization therapy of liver cancer: state of the art [J]. J Nanobiotechnology, 2023, 21(1): 42.

[16] KANG L, LI C, ROSENKRANS Z T, et al. CD38-Targeted Theranostics of Lymphoma with (89)Zr/(177)Lu-Labeled Daratumumab [J]. Adv Sci (Weinh), 2021, 8(10): 2001879.

[17] YANG E, LIU Q, HUANG G, et al. Engineering nanobodies for next-generation molecular imaging [J]. Drug Discov Today, 2022, 27(6): 1622-38.

[18] MA J, ZHANG S, YANG N, et al. Discovery of a highly specific radiolabeled antibody targeting B-cell maturation antigen: Applications in PET imaging of multiple myeloma [J]. Eur J Nucl Med Mol Imaging, 2024.

[19] WEI W, ZHANG Y, ZHANG D, et al. Annotating BCMA Expression in Multiple Myelomas [J]. Mol Pharm, 2022, 19(10): 3492-501.

[20] SONG L, JIANG S, YANG Q, et al. Development of a Novel Peptide-Based PET Tracer [(^{68})Ga]Ga-DOTA-BP1 for BCMA Detection in Multiple Myeloma [J]. J Med Chem, 2024, 67(17): 15118-30.

北京正洁检测技术有限公司
北京经济技术开发区凉水河二街5号
E-mail: ZENTest01@126.com
Tel:010-67870918

报告编号：23102301

190121340923

检 测 报 告

Test Report

委托单位：北京大学第一医院

地　　址：北京市西城区西什库大街8号

受检单位：北京大学第一医院

地　　址：北京市西城区西什库大街8号

检测区域：核医学科放射性药物实验室（G层）

检测日期：2023年11月28日

北京正洁检测技术有限公司

Beijing ZEN Testing Technology Co., Ltd.

北京正洁检测技术有限公司
北京经济技术开发区凉水河二街5号
E-mail: ZENTest01@126.com
Tel:010-67870918

23102301

190121340923

声 明

1、报告无“检测报告专用章”无效；

2、复制报告未重新加盖“检测报告专用章”无效；

3、不完整复印或部分复印本报告无效；

4、报告无编制、审核、批准签字无效；

5、报告涂改无效；

6、委托检验仅对本次检测负责；

7、对检测报告若有异议，应于收到检测报告之日起十五日内向本单位提出。

北京正洁检测技术有限公司
北京经济技术开发区凉水河二街5号
E-mail: ZENTest01@126.com
Tel:010-67870918

23102301

一、检测工作概述及结论

受检单位		北京大学第一医院
检测区域		核医学科放射性药物实验室（G层）
检测	状态	静态
	项目	风量（换气次数）风速、静压差、悬浮粒子数、温度、相对湿度、噪声、照度、沉降菌。
	依据	《洁净室施工及验收规范》GB50591-2010<br>《医药工业洁净室（区）悬浮粒子的测试方法》GB/T16292-2010<br>《医药工业洁净室（区）沉降菌的测试方法》GB/T16294-2010
判定依据		《洁净室施工及验收规范》GB50591-2010<br>《药品生产质量管理规范》（2010年修订）

检测仪器名称及型号编号			
风量罩	FL-1/ZEN-YQ049（I106138190604）	尘埃粒子计数器	ND-6100/S/ZEN-YQ066（951001091）
数字压力计	5815/ZEN-YQ072（T58151951005）	声级计	HS5633B/ZEN-YQ073（2020131843）
照度计	1339/ZEN-YQ075（191204406）	温湿度计	1260/ZEN-YQ076（190200213）
风速仪	9535-A/ZEN-YQ071（9535A1912010）	/	/

本次检测区域内的各项数据均合格，具体检测数据见分项报告。

检测报告专用章

（印章：北京正洁检测技术有限公司 检测报告专用章）

编 制	校 对	批 准	报 告 日 期
赵奔奔	吴南	石永林	2023年12月01日
（签名）赵奔奔	（签名）	（签名）石永林	

北京正洁检测技术有限公司
北京经济技术开发区凉水河二街5号
E-mail: ZENTest01@126.com
Tel:010-67870918

23102301

二、检测数据

房间名称				标记室	
洁净度等级				C级	
房间体积				8.64m²×2.6m	
检测项目	单位	合格标准		检测结果	
风量（换气次数）	次/h	≥25		48.3	
静压差	Pa	≥10		Y-01，对非洁净区	16.6
悬浮粒子数	pc/m³ (95%UCL)	0.5μm	≤352000	69459	
		5.0μm	≤2900	483	
温度	℃	18～26		19.5	
相对湿度	%RH	45～65		52.0	
噪声	dB（A）	≤60		56.4	
照度	lx	≥300		416	
沉降菌	cfu/皿·30min	≤3		0.5	
结论				合格	
备注				无	

北京正洁检测技术有限公司
北京经济技术开发区凉水河二街5号
E-mail：ZENTest01@126.com
Tel:010-67870918

23102301

房间名称				密闭式百级净化通风柜
设备编号				H1C020202007001
洁净度等级				A级
检测项目	单位	合格标准		检测结果
风速	m/s	0.36～0.54		0.43
悬浮粒子数	pc/m³（95%UCL）	0.5μm	≤3520	247
		5.0μm	≤20	8
噪声	dB（A）	≤65		59.5
照度	lx	≥300		589
沉降菌	cfu/皿·30min	＜1		0
结论				合格
备注				无

北京正洁检测技术有限公司
北京经济技术开发区凉水河二街5号
E-mail: ZENTest01@126.com
Tel:010-67870918

23102301

三、图纸

图纸	核医学科放射性药物实验室（G层）测点分布图

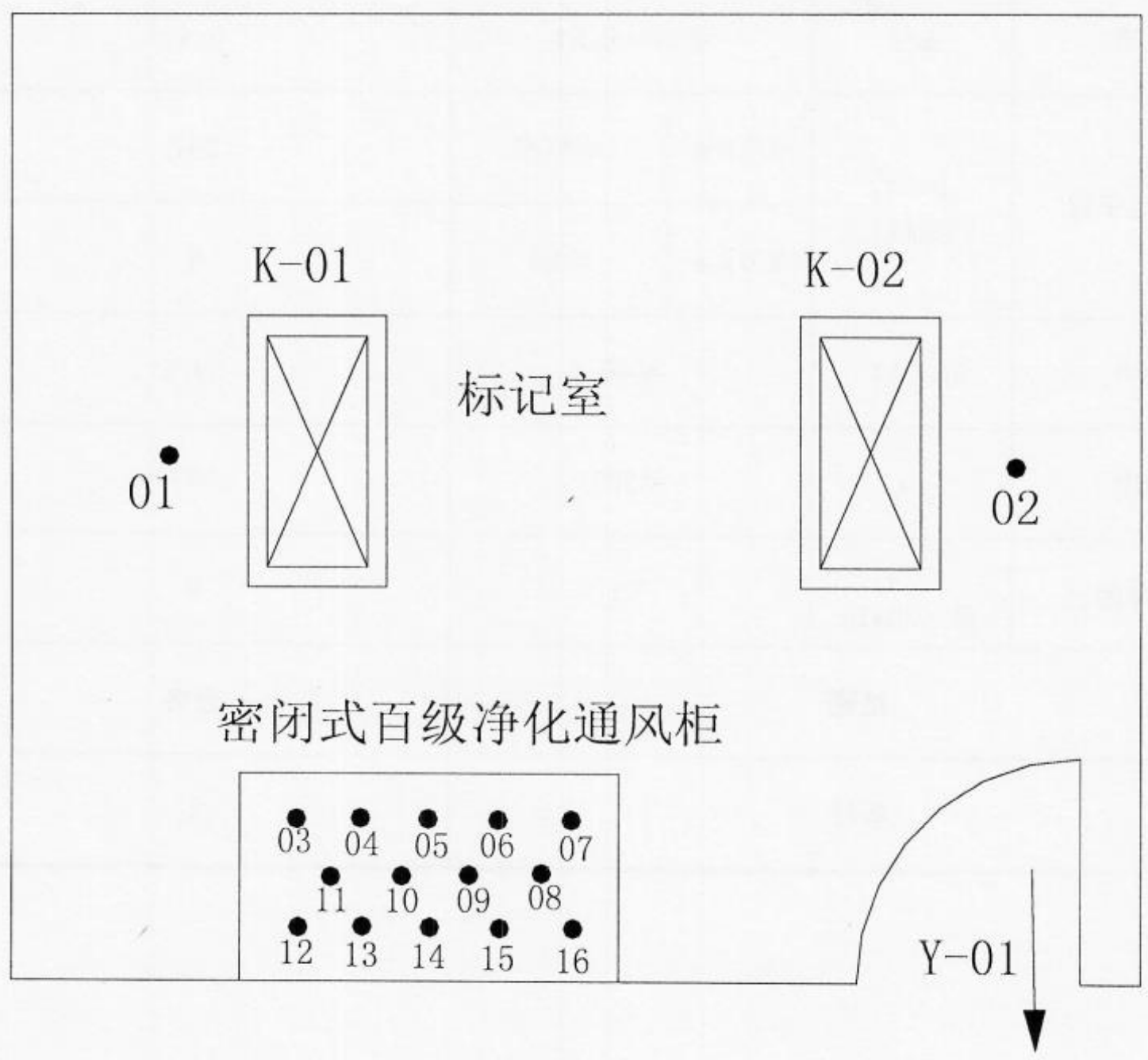

____以下空白____
