# Supplementary material for: A single-arm prospective phase I trial of 68Ga-PFBC01 PET/CT for multiple myeloma B cell maturation antigen imaging
Source: J Clin Invest. 2026 Jun 30;136(18):e207391. doi: 10.1172/JCI207391 (PMC13574143; doi:10.1172/JCI207391)
Supplement: Supplementary file 4 [file jci-136-207391-s011.pdf]

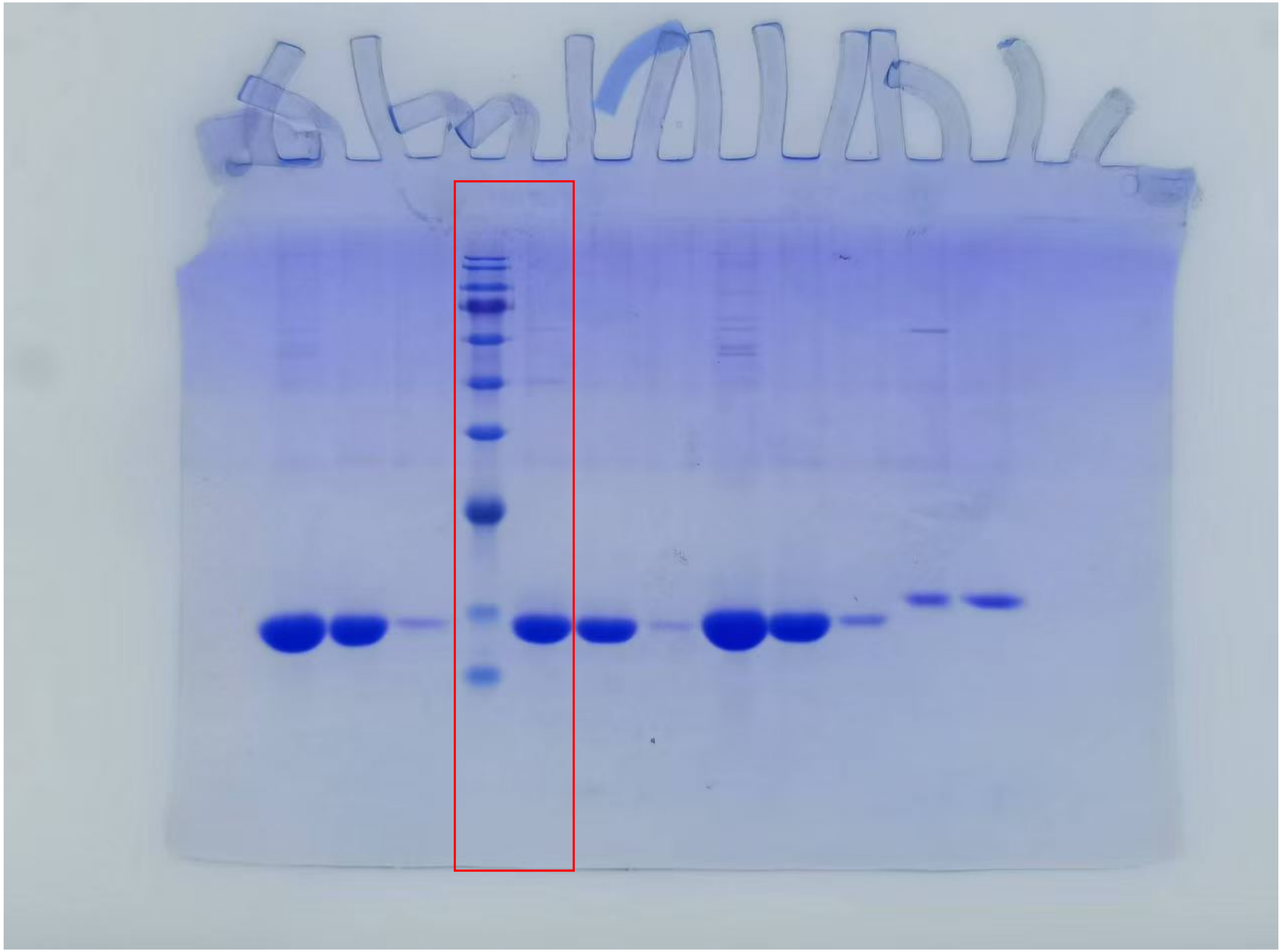

Full unedited gel for Figure 1B
